# The stochastic thermodynamics of a rotating Brownian particle in a gradient flow

**DOI:** 10.1038/srep12266

**Published:** 2015-07-21

**Authors:** Yueheng Lan, Erik Aurell

**Affiliations:** 1 The Department of Physics Tsinghua University, 100084 Beijing, China; 2 Collaborative Innovation Center of Quantum Matter, Beijing 100084, China; 3 Dept. Computational Biology and ACCESS Linnaeus Centre, KTH-Royal Institute of Technology, 106 91 Stockholm, Sweden; 4 Dept. Applied Physics and Dept. Information and Computer Science, Aalto University, Espoo, Finland

## Abstract

We compute the entropy production engendered in the environment from a single Brownian particle which moves in a gradient flow, and show that it corresponds in expectation to classical near-equilibrium entropy production in the surrounding fluid with specific mesoscopic transport coefficients. With temperature gradient, extra terms are found which result from the nonlinear interaction between the particle and the non-equilibrated environment. The calculations are based on the fluctuation relations which relate entropy production to the probabilities of stochastic paths and carried out in a multi-time formalism.

Motion of micro-sized particles in a non-equilibrium environment has recently inspired interest among researchers in diverse fields of science and engineering^1^,^2^,^3^,^4^,^5^,^6^. Interesting observables, such as diffusion constant, correlation length and various transport coefficients, are measured or computed to understand such processes. In all this characterization, entropy production is a key to the study of energy dissipation, which however could not be fully described by the commonly used over-damped Langevin description^7^. The interaction of the inertia of the particle with the non-equilibrated surroundings has to be carefully taken into account.

The change of entropy per unit time in a macroscopic fluid due to dissipative processes is a quantity defined when the fluid is close to local thermal equilibrium and which depends on gradients of intensive quantities such as temperature and local (mean) velocity^8^,^9^. On the other hand, entropy production in the environment has emerged as a fundamental quantity in mesoscopic physics underlying fluctuation relations which hold both close to and far from equilibrium^10^,^11^,^12^. This entropy production is mathematically the logarithm of the ratio of the probabilities to observe a forward and reversed system trajectory, and is therefore a functional of the whole system history^13^,^14^,^15^. The modern notion of far-from-equilbrium entropy production should be put into relation with the traditional near-to-equilibrium concept, and a first step in this direction was taken in Ref. 7, where it was shown that for an overdamped Brownian particle in a changing environment there is an “anomalous” contribution to the entropy production which reads


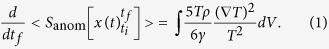


where *x*(*t*) is the particle trajectory, *ρ* the number density, *T* the temperature and *γ* the friction coefficient. Anomalous means that this contribution cannot be referred to a functional of the overdamped motion in space, and therefore represents an entropy production which belongs to the surrounding medium. Eq. (1) agrees with the normal form of the entropy production in a fluid at rest in a temperature gradient, as given in Eq. 49.6 of Ref.  9, with a mesoscopic thermal conductivity 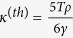. Physically, the Brownian particle performs a looping motion on a fast time scale and thereby assists the fluid in moving heat from hotter to colder regions, hence contributing to the dissipation and increasing the total disorder^7^. In this contribution we extend these results in two directions. First we show that if fluid has a spatially varying mean velocity then there is an anomalous entropy production which for a point particle and in a constant temperature field reads


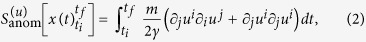


where *m* is the mass of the diffusing particle. Eq. (2), as the last two terms in Eq. 49.6 of Ref. 9, represents hydrodynamic dissipation. With temperature gradient, additional terms (shown later) are obtained which goes beyond those of Eq. 49.6 of Ref. 9 and reflects the nonlinear interaction of the particle with the fluid. Secondly we show that a Brownian particle of finite extent and having rotational degrees of freedom generates additional anomalous entropy production taking the same functional form as (1). If the particle is spherical (angular rotation frictional matrix and moment of inertia tensor both proportional to identity) then the new contribution to the thermal conductivity is 
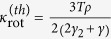
 where *γ*_2_ is an effective angular friction coefficient.

The technique used to establish these results is asymptotic expansions using the multi-time formalism^16^,^17^,^18^. To arrive at a non-trivial dependence on mean flow, *i.e.* the result (2), we need to take an advection-diffusion limit which mixes a conservation law on a faster time scale with proper dissipative action on a longer time scale, for earlier uses of analogous techniques in other contexts, *see*^19^,^20^,^21^. If the effects of mean flow are ignored, which we can mainly do for rotation, we take the overdamped limit, as in Ref. 7. The more general case, a rotating non-symmetric object coupled to a mean flow in particular, are computationally somewhat involved, and hence presented in Supplementary Information.

## Results

### Dimensions, time scales and basic equations

In the framework of Stochastic Thermodynamics it is assumed that the time scales of the surrounding fluid are much faster than those of the object. The translational degrees of freedom obey the Kramers-Langevin equations


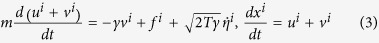


where *f*^* i*^ is an external force (protocol), *u* is the mean flow, *T* is the temperature in units such that *k*_*B*_ = 1, and *γ* is a friction coeffient. Without mean flow the rotations of the body are described by Euler equations supplemented by angular friction and angular noise^2^,^3^


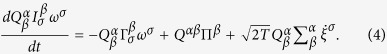


where *ω* is the angular velocity in a coordinate system fixed in the body, Π is an external torque, *I* and Γ are the moment of inertia tensor and rotational friction matrix, 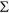 the strength of angular momentum noise, and 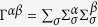. *Q* is the rotation matrix from the body frame of reference to the laboratory. The noise sources are assumed delta-correlated. As we will see it is a consistent approximation to ignore the effects of mean flow on rotation. The effects of combining rotation with a non-isotropic mobility tensor (*γ* in (3) promoted to a matrix) will be reported elsewhere.

To give numbers, assume the Brownian particle to be something like a polystyrene ball of radius *R* ~ 1 *μm*, mass *m* ~ 4.2 × 10^−12^ *g* moving in water near room temperature. Several different time scales exist across the microscopic and the mesoscopic regimes. The shortest is the microscopic collision time which is *t*_*c*_ ~ 10^−12^ *s* while the shortest time we will be interested in is the momentum relaxation time, which we can estimate from Stoke’s law to be about *t*_*r*_ ~ 10^−7^ *s*. The relaxation time and the thermal velocity of the object 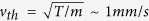 combine to give a small spatial scale 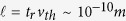. We assume that temperature, friction coefficients, external force and external torque as well as (see below) mean flow vary on a spatial scale *L* which is much larger than 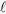. Their ratio 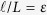 is then a small parameter. The diffusion coefficient 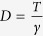 would be on the order of or less than 1(*μm*)^2^/*s* giving a slow (diffusive) time scale *L*^2^/*D*. The potential of the external force is assumed comparable to thermal energy, which implies *f* ~ *T*/*L*. To include rotations we first note that the relaxation of angular velocity happens on the same time scale *t*_*r*_ since *I* ~ *mR*^2^ and Γ ~ *γR*^2^ 22(The friction torque acting on a rotating sphere is conventionally estimated as 8*πR*^2^*η*). The typical (thermal) angular velocity is about 10^3^ radians/s such that the object rotates about 10^−4^ radians in a time *t*_*r*_. Orientation of the object will diffuse one radian on a time scale 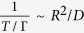 which is of the same order as it takes for the particle to move the distance of its own radius.

We here and in the following need to formally take *R* of the same order as *L* in which case the slow time scale *t*_*f*_ = *L*^2^/*D* = *R*^2^/*D* is on the order of seconds. The more realistic assumption of *R* << *L* would bring in two spatial scales (*R* and *L*) and two different long times (*R*^2^/*D* and *L*^2^/*D*), and would need to be treated by techniques such as those developed in Refs. 20,21 and 23. outside the scope of the present work.

### The advection-diffusion limit of a Brownian particle in a mean flow

We consider first the effects of mean flow and focus on a point particle (no rotation). A constant mean flow can be eliminated by a change of reference, and we can therefore further assume zero average mean flow over the large spatial scale *L*. The variation of *u* over distances *L* hence defines a characteric mean flow amplitude *u* and a corresponding time 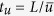. The ratio 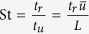 is the Stokes number of the particle in the flow, 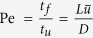 is the Péclet number, and 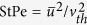. Our basic scaling assumption is that the ratios 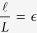 and 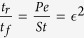 are small. The effects of the mean flow however depend on how St and Pe separately scale with 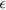.

A first possibility is the overdamped limit when St → 0 and Pe constant. The kinetic energy of the mean flow is then small compared to thermal energy and external potential energy, and there are basically no effects on the time and length scales we consider. A second possibility is Pe → ∞ and St constant which is the case of inertial particles moving in a velocity field *u*^24^,^25^. In this case diffusion can be considered weak, the anomalous entropy production terms from the fluctuating velocity would be negligible, but there would instead be entropy production terms from coarse-graining in space^26^,^27^. As noted above these are interesting questions, but outside the scope of the present work. We will here consider the third possibility when St ~ Pe^−1^, which means that the kinetic energy of the mean flow is comparable to the thermal energy and 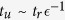. Continuing on the example above we can imagine a mean flow to be generated in the annulus between two rotating cylinders of radii *r*_−_ and *r*_+_ imparting tangential velocities *u*_−_ and *u*_+_ to the liquid. If the two cylinders have radii about 1 cm and the width of the annulus is about 1 mm the assumption of scale separation (neglecting rotation) is easily satisfied since 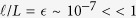. Similarly, the condition that the 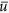 should be of the order of the thermal velocity (about 1 mm/s) of the particles means that the angular velocities of the two annuli only need to differ by about 1 rpm. The assumption that the mean flow *u* has no structure on spatial scales smaller than *L* on the other hand places a limit on how large *L* (and hence the scale separation) can be, as it supposes that a Reynolds number built on 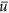 and *L* is sub-critical.

To the stochastic equations (3) corresponds a Fokker-Planck equation in a probability density *P*(*x*,*v*,*t*) over positions and velocities. In the multi-scale expansion we posit two scales in space as above and three scales in time *t*_0_ = *t*_*r*_, 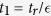 and 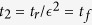, and assume that *P* depends separately on all the scales and can be expanded as


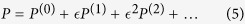


In the advection-diffusion limit *t*_1_ is comparable to *t*_*u*_. The left hand side of the the first equation in (3) can be simply written as 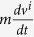 if in the right hand side we change the force *f*^* i*^ to 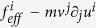, where the “effective force” 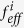 is 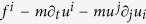. The hierarchy of equations to be solved are thus


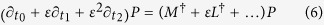


where 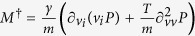 and 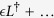 are higher-order terms. To order 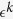 we have 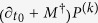 equal to terms dependent on 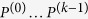 which entails the solvability condition that the right hand sides are orthogonal to functions constant in *v*^7^. To order 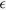 the solvability condition yields the conservation law 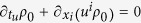, where 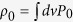. We note 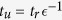, 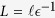, and 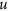 has been assumed to be on the order of 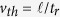 hence both sides of the conservation law are manifestly of the same order. On order 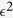 one gets that the same terms for a first order spatial density 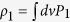, *i.e.* 
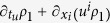
, together with diffusive terms for *ρ*_0_ vanish. These diffusive terms are, if we for the moment disregard variations of *T* and *γ* in space, 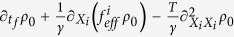 where the effective force 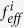 was introduced above. The combinination 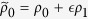 therefore obeys, up to terms of order 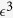, the same equation as the Fokker-Planck equation of the process


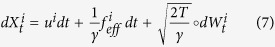


This equation explains the term “advection-diffusion limit”, where a faster process 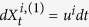 on time scale *t*_*u*_ is mixed with a slower process 
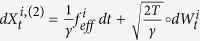
 on time scale *t*_*f*_. One may note the appearence of the Maxey terms 
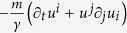
 of inertial particle theory^24^. In the case that *T* and *γ* depend on space the diffusive term in (7) should be corrected by adding the “spurious” terms 
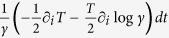
, see Refs. 28 and 7.

### The anomalous entropy production in a mean flow

In a coordinate frame at rest with the mean flow the energy of the particle is 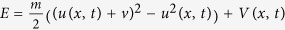, and we can define the heat *δQ* transferred from the thermal environment to the particle in a short time interval *dt* as 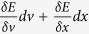. Using the Sekimoto sign convention^29^ the entropy production in the environment is then 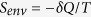 which means


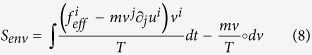


where *f*_*eff*_ was defined above. Equation (8) can also be derived as the logarithm of the ratio of the probabilities to observe a forward and reversed system trajectory defined by Eq. (3) as we show in Supplementary Information for the more general case including rotations.

Following Ref. 7 it is convenient to introduce the normalized *n*-dimensional Maxwell-Boltzmann distribution 
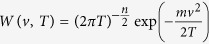
 and use 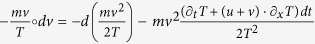 to write the functional (8) as


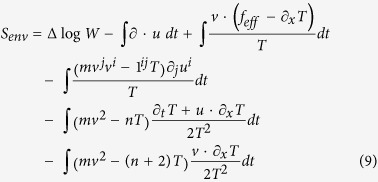


This more lengthy form reflects the eigenvector structure of *M*^†^ since 
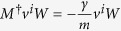
, 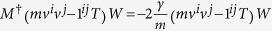 and 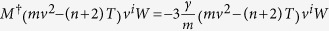. The expected entropy production *q* from some time and position in the past up to (*x*,*v*,*t*) in the present fulfills the forward Kolmogorov equation


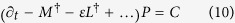


where *C* is the running cost (values at (*x*,*v*,*t*) of the integrands in (9), and the multiscale can then be carried out in an analogous manner as above. To lowest order one finds *P*_0_ = *Wq*_0_(*x*,*t*_*u*_,*t*_*f*_) and to order 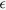 the solvability condition is 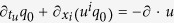 reflecting a dissipation-less entropy change by the advecting mean flow. The solution at order 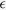 comprises the same kind of terms as above for the density and all terms in (9) (except the first two) counted with the proper eigenvalues of (*M*^†^)^−1^. On order 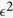 one therefore gets as solvability condition the terms of a conservation law for a first order spatial entropy function 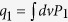, *i.e.* 
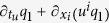
, diffusive terms for *q*_0_, and source terms from the first through fourth lines of (9). Up to terms of order 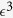 the combinination 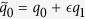 can hence be seen to obey the forward Kolmogorov equation of the expected value under the process (7) of a combined quantity 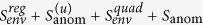 where the first term (contribution from the first line of (9) is


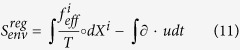


This “regular entropy production” is the canonical form of the entropy production in the first-order stochastic process (7) where the mean flow (*u*) and the overdamped force 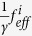 transform differently under time reversal^15^, and agrees, if taking *f* for *f*_*eff*_, with the formula derived directly in the overdamped limit in Ref. 30. The other three terms can all be called “anomalous”, as they are the remainders in the over-damped limit of an entropy production formulated for the faster time scale of under-damped dynamics. The contribution from the second line of (9) is 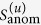 as given in (2), while the similar contribution from the third line are 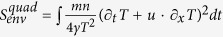, and the contribution from the fourth line of (9) are *S*_anom_ as given in (1). All the calculations presented above are straightforward though somewhat lengthy and therefore given in Supplementary Information.

We proceed to give order of magnitude estimates of 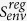, 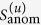, 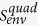 and *S*_anom_. The first term in (11) is dimensionally force times length in units of *T* which is 4 pN·nm at room temperature. It has arbitrary sign and vanishes in average in steady state, while the second term on the right-hand side of (11) can be disregarded in an incompressible fluid such as (to good enough approximation) water. The three anomalous terms are on the other hand all positive definite and hence all give contributions proportional to the duration of the process. Given that mean flow *u* is taken comparable to thermal velocity of the Brownian particle, *S*_anom_ and 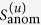 are both about 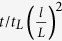 where *t*_*L*_ ~ 10^−7^ *s* is the Langevin relaxation time, *l* ~ 10^−10^ *m* is the length scale built on *t*_*L*_ and thermal velocity, and *L* is the large length scale. If we take *L* = *R* ~ 10^−6^ *m*, the particle radius, *S*_anom_ and 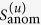 are hence about 0.1*t*, implying that there would be measurable fluctuations of the anomalous entropy production on the time scale of ten seconds if the flow (or temperature) varies at the order of micrometers. Note that the rate is inversely proportional to the square of *L* and hence quickly diminishes with the system size. If we check the particle in the Couette flow between two concentric cylinders in the above example, *δS* ~ 0.001*t* if the radius difference of the two cylinders is *L* ~ 10 *μm* while *δS* ~ 10^−7^*t* for *L* ~ 1 *mm*. In this example, the temperature is uniform in space and time, in the steady state 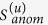 is the only term that contributes. The anomalous entropy production may be measured by a long-time observation of particle trajectories or the shear force change when adding a small dose of particles. If temperature also varies in space, the estimation becomes harder since the particle is not uniformly distributed then. Below, we carry out more precise computation in a concrete setting involving both flow and temperature gradient.

Combining all the contributions illustrated above, for a spherical particle, we are able to give the anomalous entropy production associated to the mean flow if it is irrotational


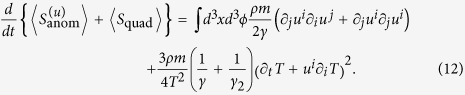


The first term on the right hand side is independent of temperature, being purely accounted for by the flow gradient. The last term results from the interaction of the fluid flow with the temperature gradient, attributed to the translational and the rotational motion of the Brownian particle.

**Figure 1:**
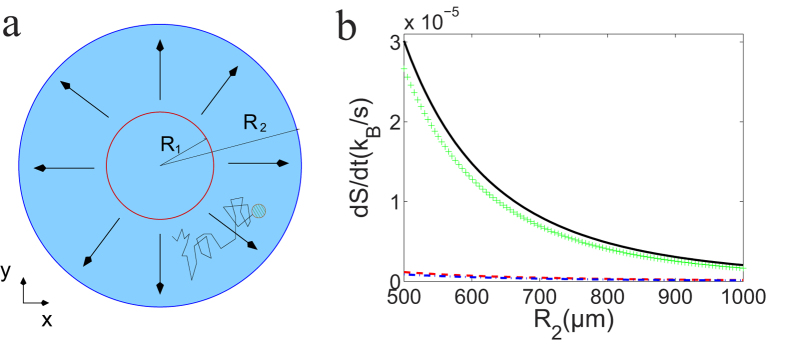
Entropy production of one Brownian particle in a gradient flow. (**a**) a ring setup for generating the gradient flow and the temperature field, (**b**) entropy contribution of different terms of Eq. (13) with increasing outer radius, coming from different sources: purely flow gradient contribution 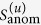 (cross) from the first term of Eq. (13); the rest two terms contributed by *S*_quad_ representing the temperature field coupled with the translation (dashed line) indicated by *γ*, the temperature field coupled with the rotation (dot-dashed line) indicated by *γ*_2_. Three contributions combined are depicted by the solid line.

In Fig. 1(a), a simple experimental setup is depicted which may be used to measure entropy production of Brownian particles in a gradient flow with temperature variation. The whole setup is rotationally symmetric with the inner radius *R*_1_ = 500 *μm* and the outer radius *R*_2_ = 1000 *μm*. Below, we use dimensionless quantities to represent our physical variables (See the previous discussion about scales of various variables. A more detailed explanation is included in the Supplementary Information.). For example, the radius of the particle is 1 which is used as the length scale and is 1 *μm* physically. The time scale is taken to be *t*_*u*_ ~ 10^−3^ *s*. With this convention, in water, the rescaled translational friction coefficient is *γ* = 4.53 × 10^3^ and the rotational one *γ*_2_ = 1.51 × 10^4^. The flow is incompressible and irrotational with a profile 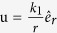 and temperature *T* = 1/(*k*_2_*r* + *t*_2_), where *k*_1_ = 2000 and *k*_2_ = 1/2000, *t*_2_ = 0.5. With this setup, Eq. (12) gives


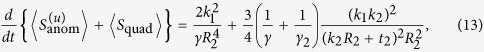


where the physical unit for the whole expression is *k*_*B*_/*t*_*u*_. Note that in Eq. (13), we did not include the contribution of *S*_anom_ which has the same order as 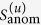. One interesting observation is that the entropy production rate is independent of the inner radius *R*_1_.

In Fig. 1(b), the contribution of different terms of Eq. (13) is depicted with increasing radius *R*_2_. It is easy to see that the first term engender most entropy production while the flow temperature mixing term only accounts for a small portion. All the rates decreases quickly with *R*_2_ but with sufficiently small *R*_2_ the entropy production seems considerable.

### The anomalous entropy production of a rotating Brownian particle

As a non-trivial illustration that macroscopic thermodynamics correctly predicts the form of mesoscopic entropy production we will now address the technically more involved case of a rotating Brownian particle with general moment of inertia and angular velocity friction operators. The friction coefficient of an object of radius *R* is by Stokes’ law of the order of *Rρ*_*f*_*v* where *ρ*_*f*_ is the density of the surrounding fluid and *v* its kinematic viscocity. If the density of the body and the fluid are not too different the relaxation time *t*_*r*_ is hence of order *R*^2^/*v*. This gives another interpretation of the scale separation *t*_*r*_/*t*_*f*_ (built on *R*) as *D*/*v*. The Stokes number is St = Re(*R*/*L*)^2^, where 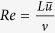 is the Reynolds number built on *L* and 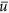, and the Péclet number is 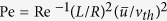. The advection-diffusion limit is therefore also given by the condition 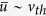 and the limit Re(*R*/*L*)^2^ → 0. We have here limited ourselves to situations where (at least formally) *R* ~ *L* which then means (as an asymptotic limit) Re → 0. At very low Reynolds numbers viscous effects are strong and the mean flow is irrotational 
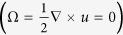
, and we can hence treat the rotating Brownian particle as if it were not influenced by the mean flow at all, except as through how *T* and Γ change in space and time. For completeness we give in Supplementary Information the full derivation including all terms which would formally appear in the advection-diffusion limit with no restriction on Re; the terms that appear here can then be found by setting Ω to zero.

Relaxation on time scale *t*_*r*_ is now described by the operator


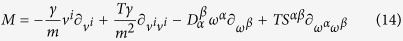


where we have introduced the angular velocity friction matrix *D* = *I*^−1^Γ and the angular velocity diffusion matrix *S* = *I*^−1^Γ*I*^−1^. These two do not necessarily commute, and it is therefore not always possible to find an orthonormal transformation (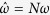, *N*^−1^ = *N*^*t*^) which simultaneously diagonalizes *D* and *S*. On the other hand, if the eigenvalues of *D* are non-degenerate *D* can always be diagonalized by a general linear transformation (*N*^−1^ not necessarily the same as *N*^*t*^). The two operators *D* and *S* act on different spaces and under a general linear transformation they transform as 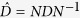 and 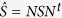. Therefore, under the further weak assumption that *D* has full rank the linear transformation *N* that diagonalizes *D* in fact also diagonalizes *S*. We show in Supplementary Information that under the stated assumptions there is a general linear transform *N* which simultaneously diagonalizes *D*, *S*, Γ and *I* under the transformations 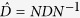, *S* = *NSN*^*t*^, 
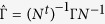
 and 
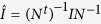
. We will from now on assume that (14) is written in this special frame 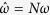 where *D* and *S* are both diagonal.

Using local charts of the orientations such that 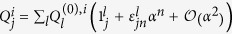
31 one can show, in analogy to (7) that the overdamped equation of motion of the orientations is


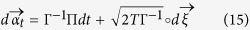


where 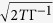 is the matrix square root, and there are no Itô or spurious corrections since Γ is constant in the body and *T* does not depend on the orientation. A globally valid description such as *e.g.* in terms of Euler angles^32^ (see Supplementary Information) will contain additional terms depending on the parametrization. The inertial term in Euler’s equation 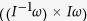 does not contribute to the overdamped equation of motion.

The entropy production in the environment of a particle following (3) and (4) is now the functional


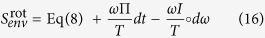


which may be rewritten as


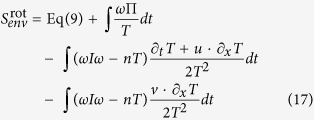


The only new terms we need to compute using the relaxation operator of (14) are 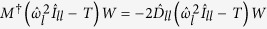 and 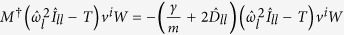 (no summation over *l* in either case) and they give terms with the same dependence on temperature gradients and mean flow as 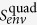 and 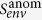 above. The second term can be written as 
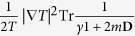
 and if the particle is spherical then 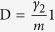 where *γ*_2_ has the dimension of a friction coefficient, which gives the simpler expression quoted in the Introduction.

Overall, without fluid flow and for a spherical Brownian particle, the anomalous entropy production could be written as


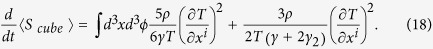


**Figure 2:**
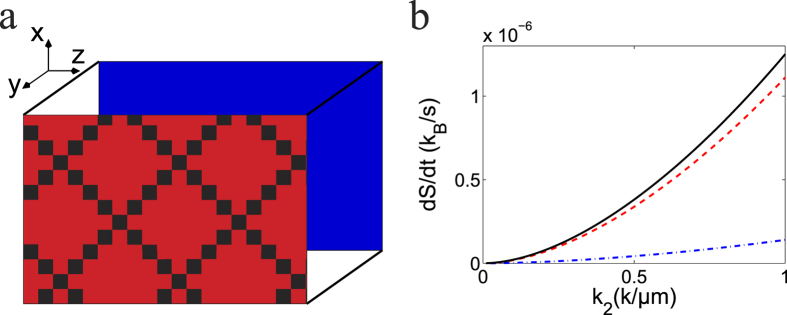
Entropy production of a spherical Brownian particle in a temperature gradient. (**a**) a box filled with water with the hot (red) and cold (blue) plate aligned in the *y*-direction; (**b**) entropy contribution of different terms of Eq. (19) with increasing temperature gradient *k*_2_ resulting from the translational motion (dashed line) indicated by *γ* in the equation and the rotational motion (dot-dashed line) indicated by *γ*_2_, two contribution combined (solid line).

In Fig. 2(a), a simple setup is used to explain a possible application of Eq. (18) where a box filled with water is in contact with two heating plate along the *y*-direction sitting at *y* = 0 and *y* = 1000 *μm*. As a result, a temperature gradient is established *T* = *k*_2_*y* + *t*_2_ with *t*_2_ = 283 *K*. As previously, a Brownian particle of radius 1 *μm* will produce an anomalous heat dissipation


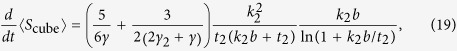


where *b* = 1000 *μm* is the distance between the two plates. In Fig. 2(b), the contribution of different degrees of freedom to entropy production is plotted against the temperature gradient. It is very clearly seen that the production rate increases quickly with the gradient. To observe such an effect in a real experiment, a large temperature gradient needs to be created. Translational motion seems to account for a major portion of the entropy production while the rotational motion produces a small portion. The reason is that *γ*_2_ is much bigger than *γ* in this case.

## Discussion

In macroscopic thermodynamics the world is divided up in two parts: the system and the thermal environment. For a fluid in a container the system is the fluid itself and all objects therein, while the thermal environment is everything else which does not remember the previous state of the system. In stochastic thermodynamics^29^, the Brownian particle plays double roles: as a mechanical system that follows Newton’s equation and as a thermal system that absorbs heat from the environment. Hence, corresponding to the external force term in the Langevin equation, a third player comes in which is the external system. It is the ideal outside control acting directly on the Brownian particle which remembers the previous state of the system but contains no thermal fluctuation. The thermal environment is the surrounding fluid which provides both the friction and the fluctuation. This tripartite division of the world is consistent with macroscopic thermodynamics if the characteristic time scale of the fluid is much smaller than that of the object and the external system, which is reasonable if the object is mesoscopic. The fluid can thus be kept close to equilibrium while the object remains far from equilibrium. The far from equilibrium entropy production in the environment in stochastic thermodynamics should then correspond to the near-equilibrium change of entropy per unit time in fluid, which is what we have found in the example of a Brownian particle with translation and rotation.

In many a situation, the over-damped Langevin equation is an excellent description of dynamics of small objects due to the ubiquitous high-friction in the mesoscopic world. The influence of the inertia term is highly suppressed by the quick momentum relaxation. However, when evaluating entropy production, which is a functional of the system history, a trace of the inertia term remains also in the limit. This anomaly is proportional to the scale separation underlying over-damped approximation, and hence small per se, but it is positive definitive, and therefore tends to overtake the entropy production from the overdamped motion as such, for sufficiently long times. In this work we have shown, in several examples, that this anomalous entropy production has the form a close-to-equilibrium entropy production in a medium, with appropriate mesoscopic transport coefficients. We believe this to be of conceptual interest, bridging the gap between entropy production in the classical macroscopic thermodynamics sense, and in the more recent stochastic thermodynamics. Such an anomalous entropy production would also lead to fluctuation relations for conditional probabilities as discussed for the simplest example in Ref. 7. In the idealized setting of our asymptotic expansion the anomalous entropy production of a single particle is on the order of 0.1 *k*_*B*_/*s* if the temperature (or flow velocity) gradient is present at the micrometer scale, and could hence be measured in comparatively short time. Recent progress in experiment enables a very precise measurement of velocities of Brownian particles^33^, which may be used in such experimental realization. In more a realistic setting, where the characteristic large length scale *L* of the process is about a hundred (or a thousand depending on the setting) times larger than the particle radius *R*, we however estimate the anomalous entropy production to be only of the order of 10^−5^ *k*_*B*_/*s* or even less, which would need much longer time (one day) of observation to be measured. The difference arises simply from two spatial derivatives which appear in all terms, and which give a *L*^−2^ scaling. Clearly more work is needed to arrive at settings where the anomalous entropy production and its fluctuations can be measured with precision.

## Methods

### Equation of motion

All the computation and analysis is based on the equation of motion of a Brownian particle immersed in a fluid with the given velocity field *u*(*x*,*t*) and subject to an external force *f*(*x*,*t*) and a torque Π which could be written as


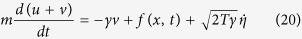



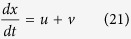



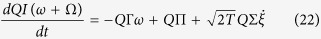



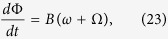


where Ω = *Q*^−1^∇ × *u*/2 is the fluid rotation, *Q* being the rotation matrix from the body to the lab frame. *v* and *ω* are the translational and the angular velocity relative to the fluid, respectively. *γ* and Γ describe frictions in the translational and the rotational movement with 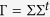. *x* is the position of the particle and Φ is the Euler angle. The matrix *B* relates the time rate of Φ to the angular velocity and *I* is the matrix of moment of inertia. *T* = *T*(*x*,*t*) is the temperature field. 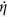 and 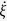 are uncorrelated Gaussian white noise. In the computation, a Fokker-Planck equation is written down corresponding to the scaled form of Eq. (23) with the particle radius *R* and the typical temperature value *T*_0_ being the basic scale for length and energy. Several characteristic time scales exist in the system including the momentum relaxation time *m*/*γ*, the thermal ballistic time 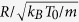 and the configuration relaxation time *R*^2^*γ*/*k*_*B*_*T*_0_. These scales are widely separated and hence could be utilized to carry out a multi-scale analysis.

### Entropy production along a trajectory

In stochastic thermodynamics, the entropy produced in the environment is associated with heat exchange with thermal reservoir while the entropy of the Brownian particle itself along a trajectory is defined^34^ as *S*_*p*_ = −ln*P*_*t*_ with *P*_*t*_ being the local density of Brownian particles. Alternatively, if the stochastic process is given by the equation


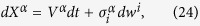


where *V*^*α*^ = *V*^*α*^(*x*,*t*) is the drift and *dw*^*i*^’s are independent Wiener processes, the environmental entropy production along a stochastic path may be given as the logarithm of the ratio of the probabilities of the forward and backward path^15^.

Both arguments give essentially identical result of the environmental entropy production


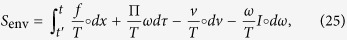


where the first two terms relate to the work done by the external force *f* and torque Π, and the last two terms to the kinetic energy associated with the translational and rotational movement. Eq. (25) could be split into three parts *S*_env_ = *S*_reg_ + *S*_quad_ + *S*_cube_ as done in Eq. (9) or Eq. (17) in the main text. Here we emphasize the analytic structure of these terms, each being a time integral of a polynomial *P*_*i*_ (*i* = reg, quad, cube). Thus, the notation *S*_cube_ is *S*_anom_ in the main text and *S*_quad_ is in fact a sum of 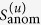 and 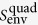. The first part is the regular entropy production seen in the over-damped Langevin equation while the last two represents anomalous contribution beyond the over-damped limit.

### Feynman-Kac formula

The Fynman-Kac formula is used to compute time averages of physical observables along trajectories of stochastic or quantum dynamics. Evaluation of the contribution of various terms in *S*_env_ starts with the generating function


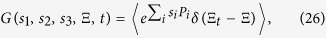


where 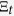 denotes the coordinate in the 12-dimensional phase space of the particle. By Feynmann-Kac formula, a linear partial differential equation of evolution type could be written down for 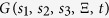
7, detailed in the Supplementary Information.

### Multi-scale analysis of the evolution equation

A multi-scale analysis is used to perturbatively solve both the Fokker-Planck equation and the equation for the generating function. With the above-mentioned time scale separation characterized by a bookkeeping small parameter 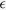, both equations could be written as 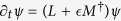, where *L*,*M* are linear operators involving derivatives of phase space variables. The multi-scale analysis is enabled by introducing two more time scales *θ*,*τ* such that 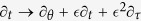 and the distribution function *ψ* is expanded as 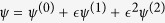. By substituting these assumptions into the evolution equation, we derive a ladder of differential equations from comparison of different orders of 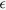. At the order 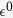, after the transient relaxation of the fastest time scales, we retrieve the local equilibrium condition. At higher orders of 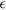, the solvability condition gives the continuity equation and the wanted equation expressed with only configuration variables.

## Additional Information

**How to cite this article**: Lan, Y. and Aurell, E. The stochastic thermodynamics of a rotating Brownian particle in a gradient flow. *Sci. Rep.* **5**, 12266; doi: 10.1038/srep12266 (2015).

## Supplementary Material

Supplementary Information
