# Supplementary material for: The stochastic thermodynamics of a rotating Brownian particle in a gradient flow
Source: Sci Rep. 2015 Jul 21;5:12266. doi: 10.1038/srep12266 (PMC4508657; doi:10.1038/srep12266)
Supplement: Supplementary Information [file srep12266-s1.pdf]

Supplementary Information: The stochastic thermodynamics of a rotating Brownian particle in a gradient flow

Yueheng Lan*[1] and Erik Aurell[2,3]

[1] *The Department of Physics, Tsinghua University, 100084 Beijing, China*
[2] *The Department of Computational Biology, AlbaNova University Centre, KTH-Royal Institute of Tenology, SE-106 91 Stockholm, Sweden*
[3] *ACCESS Linnaeus Centre, KTH-Royal Institute of Technology, SE-100 44 Stockholm, Sweden*

Miron-sized particles immersed in fluids receive extremely frequent, irregular bombarding from neighboring fluid molecules and hence execute Brownian motion - random movement in the mesoscopic scale. Kramers-Langevin type of equations are often used to describe Brownian motion which is Newton's second law plus noise terms with coefficients satisfying the fluctuation-dissipation relation due to the local thermal equilibration. In the limit of large friction or small particle mass, the inertia term may be neglected since its value is minute compared to other terms, which results in the usual Langevin equation. The trajectories of Brownian particles are very well described by this approximate equation and many physical observables can be accurately computed except the entropy production. The full Kramers-Langevin equation gives extra terms in the expression of entropy production which are not included in the usual Langevin computation. This anomaly was first shown in [1] for a Brownian particle with translational degrees of freedom. The computation here shows that rotation as well as the average flow gives additional terms to the anomalous entropy production.

This appendix is organized as follows. In section I, the equation of motion for a Brownian particle with both the translational and rotational degrees of freedom is written down and non-dimensionalized with the characteristic scales of the system. The entropy production for a general stochastic process is defined in section II, where an expression of entropy production along a stochastic path for the full Kramers-Langevin equation is given both from an intuitive physical consideration and through calculating the ratio of probabilities of the backward and forward paths. The over-damped Langevin equation in the large friction limit is derived in section III through a multi-time-scale analysis. The resulting equation predicts a different entropy production from that of the full Kramers-Langevin equation. To compute the average entropy production rate over all paths with fixed initial and terminal space-time coordinates, in section IV, a Fokker-Planck equation for the generating function of the entropy production is derived based on the Feynman-Kac formula. A similar multi-scale analysis enables derivation of an analytic expression for the average entropy production rate. The physical significance and its relevance to other work is discussed in section V.

I. EQUATION OF MOTION

A. The Kramers-Langevin description

If a Brownian particle immersed in a fluid flowing with the given velocity field $u(x,t)$ is subject to an external force $f(x,t)$, the equation of motion can be written as

$$m\frac{d(u^i+v^i)}{dt} = -\gamma v^i + f^i(x,t) + \sqrt{2T\gamma}\dot{\eta}^i \tag{1}$$

$$\frac{dx^i}{dt} = u^i + v^i \tag{2}$$

$$\frac{dQ^{\alpha\beta}I^{\beta\sigma}(\omega^\sigma+\Omega^\sigma)}{dt} = -Q^{\alpha\beta}\Gamma^{\beta\sigma}\omega^\sigma + Q^{\alpha\beta}M^\beta(x,t) + \sqrt{2T}Q^{\alpha\beta}\Sigma^{\beta\sigma}\dot{\xi}^\sigma\,, \tag{3}$$

where $\Omega = Q^{-1}\nabla\times u/2$ is the fluid rotation, Q being the rotation matrix from the body to the lab frame. For an arbitrary vector K (in the body frame), we have $Q^{-1}\dot{Q}K = (\omega+\Omega)\times K$. v^i and ω^α are the translational and the angular velocity relative to the fluid, respectively. γ is the translational friction and Γ is the rotational friction coefficient matrix. $\Gamma^{\alpha\beta} = \Sigma^{\alpha\sigma}\Sigma^{\beta\sigma}$, the Einstein summation rule being implied. Eq. (1) and (2) describe the translational motion and Eq. (3) the rotation of a Brownian particle. Different index notations are used since for translational and rotational degrees of freedom, we use different coordinate frames. x is the position of the particle and x^i is one coordinate component. $T = T(x,t)$ is the temperature field which may change slowly in space and time. I is the matrix of moment of inertia and $M^\alpha(x,t)$ describes the external torque. $\dot{\eta}^i$ and $\dot{\xi}^\beta$ are uncorrelated Gaussian white noise, *i.e.*

$$\begin{aligned}
\langle\dot{\eta}^i(t)\rangle &= \langle\dot{\xi}^\alpha(t)\rangle = 0\\
\langle\dot{\eta}^i(t)\dot{\eta}^j(s)\rangle &= \delta^{ij}\delta(s-t)\\
\langle\dot{\xi}^\alpha(t)\dot{\xi}^\beta(s)\rangle &= \delta^{\alpha\beta}\delta(s-t)\\
\langle\dot{\eta}^i(t)\dot{\xi}^\alpha(s)\rangle &= 0\,,
\end{aligned}$$

where δ^{ij} is the Kronecker delta function and $\delta(s-t)$ the Dirac delta function. The specific forms of the noise terms in Eq. (1) and Eq. (3) are used to conform to the fluctuation dissipation theorem [2].

The friction term $-\gamma v^i$ in Eq. (1) is not the most general form for the translational friction of a particle moving in a viscous fluid. For particles with arbitrary shape, the friction coefficient is a second order tensor which depends on its orientation. Strictly speaking, the scalar coefficient can be rigorously derived only for a particle with spherically symmetric surface. However, if the aspect ratio of the particle is close to one or in the long time limit, the diffusion of the particle is almost isotropic. In that case, the coefficient γ is an effective average [3–5]. For simplicity, we make this assumption in this work. For particles with arbitrary shape, there might be strong couplings between the rotation and translation or between the particle motion and fluid flow pattern. We do not consider this complication here.

In a frame moving with the particle, we have

$$\frac{du^i}{dt} = \frac{\partial u^i}{\partial t} + (u^j + v^j)\frac{\partial u^i}{\partial x^j}\,. \tag{4}$$

Eq. (1) then becomes

$$m\frac{dv^i}{dt} = -\gamma v^i + f_0^i + f_1^i + \sqrt{2T\gamma}\dot{\eta}^i\,, \tag{5}$$

where

$$f_0^i = f^i - m(\partial_t u^i + u^j\partial_j u^i) \tag{6}$$

$$f_1^i = -mv^j\partial_j u^i\,, \tag{7}$$

with ∂_j denoting $\partial/\partial x^j$. The term f_0 contains no component of v and f_1 contains the first order terms of v^j. In the body frame moving and rotating with the particle, we have

$$\frac{d\Omega^\alpha}{dt} = \frac{\partial\Omega^\alpha}{\partial t} + (u^j + v^j)\partial_j\Omega^\alpha + \dot{\phi}^\beta\partial_\beta\Omega^\alpha\,, \tag{8}$$

where ∂_β denotes $\partial/\partial\phi^\beta$. The angular variable ϕ^β describes the orientation of the Brownian particle.

To complete the equation of motion, we need another equation which relates the angular velocity in Eq. (3) to the particle orientation. If the Euler angles are used, *i.e.*, $(\phi^1\,,\phi^2\,,\phi^3) \equiv (\theta\,,\varphi\,,\psi)$, we can write

$$(\Omega^1+\omega^1\,,\Omega^2+\omega^2\,,\Omega^3+\omega^3)^t = A(\dot{\theta}\,,\dot{\varphi}\,,\dot{\psi})^t\,, \tag{9}$$

where the rotation matrix A relates changes of the Euler angles to the angular velocity components in the body coordinate

$$A = \begin{pmatrix} \cos\psi & \sin\psi\sin\theta & 0 \\ -\sin\psi & \cos\psi\sin\theta & 0 \\ 0 & \cos\theta & 1 \end{pmatrix}\,.$$

It is easy get the inverse matrix B of A

$$B = \begin{pmatrix} \cos\psi & -\sin\psi & 0 \\ \sin\psi/\sin\theta & \cos\psi/\sin\theta & 0 \\ -\sin\psi/\tan\theta & -\cos\psi/\tan\theta & 1 \end{pmatrix}\,. \tag{10}$$

With the above preparation, Eq. (3) becomes

$$I\frac{d\omega}{dt} = -\Gamma\omega + M + \Pi_0 + \Pi_1 + \Pi_2 + \sqrt{2T}\Sigma\dot{\eta}\,, \tag{11}$$

where

$$\Pi_0 = -\Omega\times(I\Omega) - I(\partial_t\Omega) - I(\partial_x\Omega)u - I(\partial_\phi\Omega)B\Omega \tag{12}$$

$$\Pi_1 = -\Omega\times(I\omega) - \omega\times(I\Omega) - I(\partial_x\Omega)v - I(\partial_\phi\Omega)B\omega \tag{13}$$

$$\Pi_2 = -\omega\times(I\omega)\,. \tag{14}$$

Here, we use directly the vector forms of physical quantities for brevity without confusion.

B. The dimensionless form

In a more realistic setting, the Brownian particle may be regarded as a polystyrene ball of radius $R \sim 1\mu m$ moving in water near the room temperature. Several different time scales exist across the microscopic and the mesoscopic regimes. The bombarding of the water molecule assumes a fast time scale $t_c \sim 10^{-12}s$. Under this frequent collision, the momentum of the particle quickly relaxes to the Maxwell-Boltzmann distribution in a time $t_r \sim 10^{-5}s$. This is also the time scale of temporal correlation of the velocity or the time scale related to the friction action. The angular momentum also changes at this relaxation time since the same molecular collisions reset the angular momentum. A significant change in the position or the orientation of the particle takes much longer time which is called the configuration relaxation time t_f. This change is caused by the Brownian motion of the particle. It takes a typical time $t_f \sim 3s$ for the above Brownian particle to move a distance of its own size R. Below, we will see that the separation of these time scales allows us using the multi-scale analysis.

Let's first scale the equation in the general setting with a temperature gradient and an external force into a dimensionless form

$$x^i \to L\tilde{x^i}\,, T \to T_0\tilde{T}\,, v^i \to \sqrt{\frac{T_0}{m}}\tilde{v^i}\,, u^i \to \sqrt{\frac{T_0}{m}}\tilde{u^i}\,, t \to \tilde{t}L/\sqrt{T_0/m}\,, f_0 \to \tilde{f_0}\frac{T_0}{L}\,, f_1 \to \tilde{f_1}\frac{T_0}{L}\,, \tag{15}$$

where L is the typical length over which the temperature varies and T_0 is the typical temperature value, say, the room temperature. The velocity is scaled by the average thermal velocity of the ball $v_{\text{therm}} = \sqrt{2T_0/m} \sim 4cm/s$, where we have taken the Boltzmann constant $k_B = 1$. Note that we have scaled the fluid velocity u^i with the same thermal velocity, which marks the magnitude of the fluid flow and also seems reasonable for a real experiment. The time variable is scaled with the thermal ballistic flight time and the force with the thermal force. If the rescaled friction coefficient is used $\tilde{\gamma}$

$$\tilde{\gamma} = \frac{L}{\sqrt{T_0}}\frac{\gamma}{\sqrt{m}}\,, \tag{16}$$

Eq. (1) becomes

$$\frac{d\tilde{v^i}}{d\tilde{t}} = -\tilde{\gamma}\tilde{v^i} + \tilde{f_0^i} + \tilde{f_1^i} + \sqrt{2\tilde{T}\tilde{\gamma}}\frac{d\eta^i}{d\tilde{t}}\,, \tag{17}$$

where variables with tilde are all dimensionless. In the large friction limit, $\tilde{\gamma}$ is large, which could help us define a super-fast time scale θ and a slow time scale τ

$$\theta : \frac{L}{\sqrt{T_0/m}}(\tilde{\gamma})^{-1} = \frac{m}{\gamma} \tag{18}$$

$$\tau : \frac{L}{\sqrt{T_0/m}}\tilde{\gamma} = \frac{L^2}{T_0/\gamma}\,. \tag{19}$$

Note that $\theta \sim m/\gamma \sim t_r$ is related to the momentum relaxation and τ to the configuration relaxation since $T_0/\gamma \sim D_{\text{conf}}$, the usual particle diffusion constant.

Below, we go on to the rescaling of the rotation equation. Here, the length scale could be conveniently chosen to be the particle radius R. Then, if we make the following choice

$$I \to mR^2\tilde{I}\,, \omega \to \frac{\sqrt{T_0/m}}{R}\tilde{\omega}\,, \Omega \to \frac{\sqrt{T_0/m}}{R}\tilde{\Omega}\,, \tilde{\Gamma} = \Gamma\frac{L}{\sqrt{mT_0}R^2}$$

$$\tilde{M} = \frac{L}{T_0R}M\,, \tilde{\Pi_0} = \frac{L}{T_0R}\Pi_0\,, \tilde{\Pi_1} = \frac{L}{T_0R}\Pi_1\,, \tilde{\Pi_2} = \frac{L}{T_0R}\Pi_2\,, \tilde{\Sigma} = \frac{1}{R}\sqrt{\frac{L}{\sqrt{mT_0}}}\Sigma\,,$$

where the component-denoting superscripts of variables have been omitted for brevity, Eq. (3) becomes

$$\tilde{I}\frac{d\tilde{\omega}}{d\tilde{t}} = -\tilde{\Gamma}\tilde{\omega} + \tilde{M} + \tilde{\Pi_0} + \tilde{\Pi_1} + \tilde{\Pi_2} + \sqrt{2\tilde{T}}\tilde{\Sigma}\dot{\tilde{\xi}}\,. \tag{20}$$

Under the given scaling Eq. (15) and (20), the other two equations Eq. (2) and (9) become

$$\frac{d\tilde{x}}{d\tilde{t}} = \tilde{u} + \tilde{v} \tag{21}$$

$$\frac{d\phi}{d\tilde{t}} = \frac{L}{R} B(\tilde{\Omega} + \tilde{\omega}) \,. \tag{22}$$

We now estimate the size of $\tilde{\Gamma}$ and $\tilde{M}$

$$\tilde{\Gamma} \sim \Gamma \frac{L}{\sqrt{mT_0}R^2} \sim \gamma R^2 \frac{L}{\sqrt{mT_0}R^2} = \frac{1}{m/\gamma}\frac{L}{\sqrt{T_0/m}} = \frac{L\gamma}{\sqrt{T_0 m}} \,,$$

which is of similar size to $\tilde{\gamma}$. Here we used the angular friction estimation $\Gamma \sim \gamma R^2$. For $\tilde{M}$, we have

$$\tilde{M} \sim \frac{L}{T_0 R} M \sim \frac{L}{T_0 R} T_0 \sim \frac{L}{R} \,,$$

if we assume the size of the external torque $M \sim T_0$ similar to the thermal torque. It is easy to check that $\Pi_{0,1,2} \sim T_0$.

Note that up to now, we have two spatial scales: R, the size of the diffusing ball and L, the characteristic length of the temperature variation. Usually, $L \gg R$, which implies two different configuration change time scales $t_1 \sim R^2/D_{\text{therm}} \sim t_f$ and a much larger one $t_2 \sim L^2/D_{\text{therm}}$. If $L \sim 10^3 R$, then $t_2 \sim 10^6 t_1$! This will introduce a very long time scale and is of course reasonable since diffusion over a macroscopic distance takes much longer than over a mesoscopic distance. However, if we mainly care about the mesoscopic time scale separation, we could use R instead of L in the scaling equation Eq. (15) and (16), which just means that we use a smaller time and space unit to measure the motion. The long time evolution of the system is gradually unfolded through progression with the equation of motion. Below, we just use one spatial scale R.

C. A very general assumption of the friction matrix

For notational brevity, we omit the tilde over symbols. Even in the most general case, the moment of inertia I and the friction coefficient Γ should be symmetric, i.e., $I^t = I \,, \Gamma^t = \Gamma$. Let's assume that matrix I has an inverse J, $IJ = \mathbb{I}$, where $\mathbb{I}$ denotes the identity matrix. Then also $J^t = J$. We assume that matrix N diagonalizes the product $J\Gamma$,

$$NJ\Gamma\bar{N} = D \equiv \text{Diag}(d^1 \,, d^2 \,, \cdots \,, d^n) \,,$$

where the notation $\bar{N} = N^{-1}$ is used. Note that we do NOT assume $J\Gamma = \Gamma J$ and hence the matrix N may not be symmetric. The angular part of the equation of motion then becomes

$$\frac{d\hat{\omega}}{dt} = -D\hat{\omega} + \hat{J}\hat{\Pi} + \sqrt{2T}\hat{J}N\Sigma\dot{\xi}$$

$$\frac{d\hat{\Phi}}{dt} = \hat{B}(\hat{\omega} + \hat{\Omega}) \,, \tag{23}$$

where

$$\hat{\omega} = N\omega \,, \hat{J} = NJ\bar{N} \,, \hat{I} = NI\bar{N} \,, \hat{\Pi} = N\Pi$$
$$\hat{\Phi} = N\Phi \,, \hat{B} = NB\bar{N} \,, \hat{\Omega} = N\Omega$$
$$\hat{\Pi} = \hat{M} + \hat{\Pi}_0 + \hat{\Pi}_1 + \hat{\Pi}_2 = N(M + \Pi_0 + \Pi_1 + \Pi_2)$$
$$\hat{\Pi}_0^\alpha = -N^{\alpha\beta}\varepsilon^{\beta\mu\nu}\bar{N}^{\mu\mu'}\bar{N}^{\nu\nu'}\hat{\Omega}^{\mu'}\hat{I}^{\nu'\sigma}\hat{\Omega}^\sigma - \hat{I}^{\alpha\beta}\partial_t\hat{\Omega}^\beta - \hat{I}^{\alpha\beta}\partial_k\hat{\Omega}^\beta u^k - \hat{I}^{\alpha\beta}\partial_\sigma\hat{\Omega}^\beta\hat{B}^{\sigma\mu}\hat{\Omega}^\mu$$
$$\hat{\Pi}_1^\alpha = -N^{\alpha\beta}\varepsilon^{\beta\mu\nu}\bar{N}^{\mu\mu'}\bar{N}^{\nu\nu'}\hat{\Omega}^{\mu'}\hat{I}^{\nu'\sigma}\hat{\omega}^\sigma - N^{\alpha\beta}\varepsilon^{\beta\mu\nu}\bar{N}^{\mu\mu'}\bar{N}^{\nu\nu'}\hat{\omega}^{\mu'}\hat{I}^{\nu'\sigma}\hat{\Omega}^\sigma - \hat{I}^{\alpha\beta}\partial_k\hat{\Omega}^\beta v^k - \hat{I}^{\alpha\beta}\partial_\sigma\hat{\Omega}^\beta\hat{B}^{\sigma\mu}\hat{\omega}^\mu$$
$$\hat{\Pi}_2^\alpha = -N^{\alpha\beta}\varepsilon^{\beta\mu\nu}\bar{N}^{\mu\mu'}\bar{N}^{\nu\nu'}\hat{\omega}^{\mu'}\hat{I}^{\nu'\sigma}\hat{\omega}^\sigma \,,$$

where $\varepsilon^{\beta\mu\nu}$ is the conventional Levi-Civita symbol and $\partial_\sigma \equiv \partial/\partial\hat{\phi}^\sigma$.

Based on Eqs. (17 ,21 ,23), we may write the Fokker-Planck equation for the probability density $p = p(t, x, \hat{\phi}, v, \hat{\omega})$

$$\begin{aligned}\partial_t p + \partial_i((u^i + v^i)p) + \partial_\alpha(\hat{B}^{\alpha\beta}(\hat{\Omega}^\beta + \hat{\omega}^\beta)p) + \frac{\partial}{\partial v^i}((f_0^i + f_1^i)p) - \gamma\frac{\partial}{\partial v^i}(v^i p) - D^{\alpha\beta}\frac{\partial}{\partial\hat{\omega}^\alpha}(\hat{\omega}^\beta p) \\ + \hat{J}^{\alpha\beta}\frac{\partial}{\partial\hat{\omega}^\alpha}(\hat{\Pi}^\beta p) = T\gamma\frac{\partial^2 p}{\partial v^i \partial v^i} + T(\hat{J}N\Gamma N^t\hat{J}^t)^{\alpha\beta}\frac{\partial^2 p}{\partial\hat{\omega}^\alpha\hat{\omega}^\beta}\,.\end{aligned} \tag{24}$$

It is convenient to use the notation $S = \hat{J}N\Gamma N^t\hat{J}^t$, $F = (NN^t)^{-1}$. We may prove the lemma below

Lemma I.1 *If no diagonal terms of the diagonal matrix D are equal, the matrices S and $F\hat{I}$ are all diagonal.*

Proof: It is easy to check that $F\hat{I}S = SF\hat{I} = D$ by a direct computation

$$\begin{aligned}F\hat{I}S &= (NN^t)^{-1}\hat{I}\hat{J}N\Gamma N^t\hat{J}^t = \bar{N}^t\bar{N}N\Gamma N^t\bar{N}^t J^t N^t \\ &= \bar{N}^t\Gamma J^t N^t = (NJ\Gamma^t\bar{N})^t = (NJ\Gamma\bar{N})^t = D^t = D\,, \\ SF\hat{I} &= \hat{J}N\Gamma N^t\hat{J}^t(NN^t)^{-1}\hat{I} = NJ\bar{N}N\Gamma N^t\bar{N}^t J^t N^t\bar{N}^t\bar{N}NI\bar{N} \\ &= NJ\Gamma J^t I\bar{N} = NJ\Gamma\bar{N} = D\,,\end{aligned}$$

which implies that $F\hat{I} = \bar{S}D = D\bar{S}$ where $\bar{S} = S^{-1}$. If the diagonal entries of D are not equal, then $\bar{S}$ should be diagonal. As a result, the matrices S and $F\hat{I}$ are all diagonal.

Later we will use the matrix H whose entry is defined by

$$H^{\alpha\beta} = \frac{2S^{\alpha\beta}}{d^\alpha + d^\beta}\,, \tag{25}$$

which is also diagonal if the conditions in Lemma I.1 are satisfied. We may prove a second lemma

Lemma I.2 *The matrix H is the inverse of $F\hat{I}$,* i.e., $F\hat{I} = H^{-1}$.

Proof: From $F\hat{I} = \bar{S}D = D\bar{S}$, we have $S = (F\hat{I})^{-1}D = D(F\hat{I})^{-1}$

$$\begin{aligned}S^{\alpha\beta} &= \frac{((F\hat{I})^{-1}D)^{\alpha\beta} + (D(F\hat{I})^{-1})^{\alpha\beta}}{2} \\ &= \frac{((F\hat{I})^{-1})^{\alpha\beta}d^\beta + d^\alpha((F\hat{I})^{-1})^{\alpha\beta}}{2} = \frac{((F\hat{I})^{-1})^{\alpha\beta}(d^\beta + d^\alpha)}{2}\,,\end{aligned}$$

since D is diagonal. Therefore

$$H^{\alpha\beta} = \frac{2S^{\alpha\beta}}{d^\alpha + d^\beta} = ((F\hat{I})^{-1})^{\alpha\beta}\,.$$

Therefore, we have $F\hat{I}H = \mathbb{I}$.

II. THE ENTROPY PRODUCTION IN A STOCHASTIC PROCESS

In equilibrium thermodynamics, entropy is related to the release and absorption of heat. In non-equilibrium statistical physics, entropy production is a measure of irreversibility of the dynamics. In stochastic thermodynamics, for any stochastic process defined by the equation

$$dX^\alpha = V^\alpha dt + \sigma_i^\alpha dw^i\,, \tag{26}$$

where $V^\alpha = V^\alpha(x,t)$ is the drift and dw^i's are independent Wiener processes, we may define entropy production along a stochastic path as the logarithm of the ratio of the probabilities of the forward and

the backward path [6], if a reversing protocol is properly chosen. For brevity, we directly write down the environmental entropy production

$$\hat{S}_{\text{env}} = 2\int \hat{V}_+^\alpha g_{\alpha\beta}(\circ dX^\beta - V_-^\beta dt) - \int \partial_\alpha V_-^\alpha dt\,, \tag{27}$$

where $\hat{V}^\alpha = V^\alpha - \frac{1}{2}\partial_\gamma g^{\alpha\gamma}$ and the plus and the minus sign in the subscript denote the choice of the time irreversible and the time reversible part of the velocity field. The matrix $g_{\alpha\beta}$ is the inverse of $g^{\alpha\beta} \equiv \sum_i \sigma_i^\alpha \sigma_i^\beta$. Eq. (27) will be used directly below for the environment entropy production in the over-damped limit.

As an example, under the Kramers-Langevin dynamics given by Eq. (1), (2), (3) and (23), the entropy production may be computed directly by calculating the probabilities of the forward trajectory $(x_{[t',t]}\,, \phi_{[t',t]}\,, v_{[t',t]}\,, \omega_{[t',t]})$ and the backward trajectory $(x_{[t,t']}\,, \phi_{[t,t']}\,, -v_{[t,t']}\,, -\omega_{[t,t']})$ in the phase space. We will see that the result matches our physical intuition straightforwardly.

In a short time denoted by $\Delta = t - t'$, the solution of Eq. (24) can be written as

$$\begin{aligned} p(x\,,\phi\,,v\,,\omega\,,t|x'\,,\phi'\,,v'\,,\omega'\,,t') =&\delta(x - x' - (\overline{u} + \overline{v})\Delta)\delta(\phi - \phi' - \overline{B}(\overline{\Omega} + \overline{\omega})\Delta) \\ \frac{\exp\left(-\frac{(v-v'-(\overline{f_0}+\overline{f_1})\Delta+\gamma\overline{v}\Delta)^2}{4\overline{T}\gamma\Delta} + \frac{n}{2}\gamma\Delta\right)}{(4\pi\overline{T}\gamma\Delta)^{n/2}} & \frac{\exp\left(-\frac{(\omega-\omega'-J\Pi\Delta+D\overline{\omega}\Delta)^\alpha \bar{S}^{\alpha\beta}(\omega-\omega'-J\Pi\Delta+D\overline{\omega}\Delta)^\beta}{4\overline{T}\Delta} + \frac{\Delta}{2}d_{ii} + \frac{\Delta}{2}(\partial_\alpha\Omega^\beta)\overline{B}^{\alpha\beta}\right)}{(4\pi\overline{T}\Delta)^{n/2}(\text{Det}(S))^{1/2}}\,, \end{aligned} \tag{28}$$

where $d_{ii} = \text{Trace}(D)$ and $\bar{S} = S^{-1}$. The overbars over other symbols denote evaluation at the mid point

$$\overline{v} = \frac{v+v'}{2}\,, \overline{u} = \frac{u(x,t)+u(x',t')}{2}\,, \overline{\Omega} = \frac{\Omega(x,t)+\Omega(x',t')}{2}\,, \overline{\omega} = \frac{\omega+\omega'}{2}\,, \overline{f_{0,1}} = f_{0,1}(\overline{x}\,,\overline{t})\,, \overline{T} = T(\overline{x})\,.$$

The reversed path on the same short time interval has a probability

$$\begin{aligned} p(x'\,,\phi'\,,-v'\,,-\omega'\,,t|x\,,\phi\,,-v\,,-\omega\,,t') =&\delta(x - x' - (\overline{u} + \overline{v})\Delta)\delta(\phi - \phi' - \overline{B}(\overline{\Omega} + \overline{\omega})\Delta) \\ \frac{\exp\left(-\frac{(v-v'-(\overline{f_0}+\overline{f_1})\Delta-\gamma\overline{v}\Delta)^2}{4\overline{T}\gamma\Delta} + \frac{n}{2}\gamma\Delta\right)}{(4\pi\overline{T}\gamma\Delta)^{n/2}} & \frac{\exp\left(-\frac{(\omega-\omega'-J\Pi\Delta-D\overline{\omega}\Delta)^\alpha \bar{S}^{\alpha\beta}(\omega-\omega'-J\Pi\Delta-D\overline{\omega}\Delta)^\beta}{4\overline{T}\Delta} + \frac{\Delta}{2}d_{ii} - \frac{\Delta}{2}(\partial_\alpha\Omega^\beta)\overline{B}^{\alpha\beta}\right)}{(4\pi\overline{T}\Delta)^{n/2}(\text{Det}(S))^{1/2}}\,. \end{aligned} \tag{29}$$

The ratio between the probabilities of the backward and the forward path is then

$$\frac{p(x'\,,\phi'\,,-v'\,,-\omega'\,,t|x\,,\phi\,,-v\,,-\omega\,,t')}{p(x\,,\phi\,,v\,,\omega\,,t|x'\,,\phi'\,,v'\,,\omega'\,,t')} = \exp\left((v-v')\frac{\overline{v}}{\overline{T}} - \frac{(\overline{f_0}+\overline{f_1})\overline{v}}{\overline{T}}\Delta + (\omega - \omega' - J\Pi\Delta)^\alpha \bar{S}^{\alpha\beta}\frac{(D\omega)^\beta}{\overline{T}} - \Delta(\partial_\alpha\Omega^\beta)\overline{B}^{\alpha\beta}\right)\,. \tag{30}$$

Utilizing the Markov property of the stochastic dynamics, over a whole time interval $[t'\,,t]$, this ratio becomes

$$\begin{aligned} \frac{p(x_{[t,t']}\,,\phi_{[t,t']}\,,-v_{[t,t']}\,,-\omega_{[t,t']})}{p(x_{[t',t]}\,,\phi_{[t',t]}\,,v_{[t',t]}\,,\omega_{[t',t]})} =& \exp\left(-\int_{t'}^t \frac{(f_0+f_1)^i v^i}{T}d\tau - \frac{v^i}{T}\circ dv^i - \frac{\omega^\alpha (FI)^{\alpha\beta}}{T}\circ d\omega^\beta \right. \\ &\left. + \frac{J\Pi}{T}FI\omega d\tau + (\partial_\alpha\Omega^\beta)B^{\alpha\beta}d\tau\right) \equiv e^{-\hat{S}_{\text{env}}}\,. \end{aligned} \tag{31}$$

Therefore, Eq. (31) shows the exact relation between the ratio of probabilities of the backward and forward path and the entropy increase S_{env} in the environment. Compared with S_{env} defined in Eq. (33) below, S_{env} contains a factor which is devoid of any information of the Brownian particle and is present even in the absence of noise. It is the entropy production related to the fluid flow itself and disappears for the irrotational flow ($\Omega = 0$).

On the other hand, the entropy production for this system could be written down directly based on the usual physics interpretation of the equations themselves. Let's consider the energy exchange of the particle

with the environment based on Eqs. (17 ,21 ,23). Again, for notational brevity, we omit the hat over symbols. Due to energy conservation, the heat released into the environment is

$$Q = \int_{t'}^{t} (f_0^i + f_1^i) \circ dx^i + (J\Pi)^\alpha (FI)^{\alpha\beta} \omega^\beta d\tau - v^i \circ dv^i - \omega^\alpha (FI)^{\alpha\beta} \circ d\omega^\beta \,, \tag{32}$$

where $\circ$ denotes the Stratonović product. Therefore, the entropy produced in the environment is

$$S_{\text{env}} = \int_{t'}^{t} \frac{f_0^i + f_1^i}{T} \circ dx^i + \frac{(J\Pi)^\alpha}{T} (FI)^{\alpha\beta} \omega^\beta d\tau - \frac{v^i}{T} \circ dv^i - \frac{\omega^\alpha}{T} (FI)^{\alpha\beta} \circ d\omega^\beta \,. \tag{33}$$

The entropy of the particle is [7]

$$S_p = -\ln p(x, v, \phi, \omega, t) \,, \tag{34}$$

where $P(x, v, \phi, \omega, t)$ is the solution of Eq. (24). Therefore, the total entropy change is

$$S_{\text{tot}} = -\ln p_t + \ln p_{t'} + \int_{t'}^{t} \frac{f_1^i + f_2^i}{T} \circ dx^i + \frac{(J\Pi)^i}{T} (FI)^{ij} \omega^j d\tau - \frac{v^i}{T} \circ dv^i - \frac{\omega^i}{T} (FI)^{ij} \circ d\omega^j \,. \tag{35}$$

From the above derivation, it is seen that if the stochastic equation of motion is known the ratio of probabilities associated with the forward and the backward path gives the entropy production. In the following, we will derive a reduced equation of motion for the Brownian particle in the large friction limit and calculate the entropy production in that case.

III. THE OVER-DAMPED EQUATION OF MOTION IN THE LARGE FRICTION LIMIT

In the large friction limit, the time scales are well separated so that a multi-scale analysis is feasible. Here, we follow the standard approach to derive a perturbation solution of Eq. (24). To start, we introduce a bookkeeping small parameter ϵ and make the following substitution

$$\gamma \to \epsilon^{-1}\gamma \,, D \to \epsilon^{-1} D \,, S \to \epsilon^{-1} S \,, \tag{36}$$

in Eq. (24), which results in

$$(\frac{\partial}{\partial t} - L^\dagger - \epsilon^{-1} M^\dagger) p = 0 \,, \tag{37}$$

where

$$L = (u^i + v^i) \frac{\partial}{\partial x^i} + B^{\alpha\beta} (\omega^\beta + \Omega^\beta) \frac{\partial}{\partial \phi^\alpha} + (f_0 + f_1)^i \frac{\partial}{\partial v^i} + J^{\alpha\beta} \Pi^\beta \frac{\partial}{\partial \omega^\alpha} \tag{38}$$

$$M = \gamma(-v^i \frac{\partial}{\partial v^i} + T \frac{\partial^2}{\partial v^i \partial v^i}) - D^{\alpha\beta} \omega^\beta \frac{\partial}{\partial \omega^\alpha} + T S^{\alpha\beta} \frac{\partial^2}{\partial \omega^\alpha \partial \omega^\beta} \,. \tag{39}$$

Eq. (39) is the scaled form of the operator M defined after Eq. (7) in the main text. To proceed, let's use the fast time $\theta = \epsilon^{-1} t$ and the slow one $\tau = \epsilon t$ and write the probability distribution function as an expansion over ϵ

$$p = p^{(0)} + \epsilon p^{(1)} + \epsilon^2 p^{(2)} + \cdots \,. \tag{40}$$

Substitute the expansion Eq. (40) and the mutilple time scales $\theta \,, t \,, \tau$ with $\partial_t \to \epsilon^{-1}\partial_\theta + \partial_t + \epsilon \partial_\tau$ into Eq. (37) and a hierarchy of equations may be obtained by comparing different orders of ϵ. Next, we will analyze these equations order by order, starting with the fastest time scale.

A. The momentum relaxation time scale ϵ^{-1}

The fastest time scale is the momentum relaxation time scale (ϵ^{-1}), in which we get the equation below

$$(\frac{\partial}{\partial\theta} - M^{\dagger})p^{(0)} = 0\,. \quad (41)$$

Note that the variables v^i and ω^i separate in the operator $M^{\dagger}$, so the eigenfunction is a product of the component eigenfunctions, being products of Hermite polynomials multiplied by the weight

$$W(v,\omega,T) = \frac{1}{(2\pi T)^{n/2}}e^{-\frac{v^2}{2T}}\frac{\mathrm{Det}(FI)^{1/2}}{(2\pi T)^{n/2}}e^{-\frac{\omega FI\omega}{2T}}\,, \quad (42)$$

which is the local Maxwellian distribution of the velocities. Note that now we are actually in the 'hat' frame, so in the distribution for the angular velocity, we have FI in the exponent which looks weird. If we used un-hatted variables, there would appear $\omega I\omega$ in the exponent. The temperature $T = T(x,\tau)$ is assumed to be a slowly varying function of the space and time variables. Eq. (24) will be viewed as a general diffusion equation in a phase space of dimension n. The eigenfunction corresponding to eigenvalue zero is unique and given by Eq. (42), which is the only survived component in the long time limit $\theta \to \infty$. Therefore, ignoring this transient behavior, we have

$$p^{(0)}(x\,,\phi\,,v\,,\omega\,,t\,,\tau) = \rho(x\,,\phi\,,t\,,\tau)W(v\,,\omega\,,T)\,, \quad (43)$$

where ρ is the marginal probability distribution in the configuration space.

The Maxwellian distribution Eq. (42) gives

$$\frac{\partial W}{\partial v^i} = (-\frac{v^i}{T})W\,, \frac{\partial W}{\partial \omega^\alpha} = (-\frac{(FI)^{\alpha\beta}\omega^\beta}{T})W$$
$$\frac{\partial W}{\partial x^i} = (\frac{v^2-nT}{2T^2} + \frac{\omega FI\omega - nT}{2T^2})\frac{\partial T}{\partial x^i}W\,, \frac{\partial W}{\partial \tau} = (\frac{v^2-nT}{2T^2} + \frac{\omega FI\omega - nT}{2T^2})\frac{\partial T}{\partial \tau}W\,,$$

which also leads to some simple integration results

$$\int dvd\omega W(v\,,\omega\,,T) = 1\,, \int v^i W dvd\omega = \int \omega^\alpha W dvd\omega = 0$$
$$\int dvd\omega v^i v^i W = \int dvd\omega \omega^\alpha (FI)^{\alpha\beta}\omega^\beta W = nT$$
$$\int dvd\omega v^i v^i v^j v^j W = n(n+2)T^2\,,$$

where the integration is taken over the whole space of $v\,,\omega$. Similar results may be obtained for more complex integrands. In addition, note that $W(v\,,\omega\,,T)$ is an even function of v and ω so that any integration involving odd number of $v\,,\omega$ components would be zero. We will use this property later.

B. The intermediate thermal ballistic time scale

The rescaling of time was done with the thermal ballistic time, which shows up as the intermediate scale (ϵ^0). We obtain an equation for $p^{(1)}$

$$(\frac{\partial}{\partial\theta} - M^{\dagger})p^{(1)} = -(\frac{\partial}{\partial t} - L^{\dagger})p^{(0)}\,, \quad (44)$$

which over the fast time relaxes quickly to

$$M^{\dagger}p^{(1)} = (\frac{\partial}{\partial t} - L^{\dagger})p^{(0)}\,. \quad (45)$$

Based on Eq. (45), we have the following solvability condition: the right hand side of Eq. (45) should be orthogonal to the null space of the operator M. In particular, it has to be orthogonal to a constant (not depending on v and ω). Integrating Eq. (45) over v and ω, most terms vanish due to the reflection symmetry, which leads to

$$\frac{\partial \rho}{\partial t} + \frac{\partial}{\partial x^i}(u^i \rho) + \frac{\partial}{\partial \phi^\alpha}(B^{\alpha\beta}\Omega^\beta \rho) = 0\,, \tag{46}$$

which is the continuity equation for the density ρ. Henceforth,

$$\begin{aligned} M^\dagger p^{(1)} &= (\frac{\partial}{\partial x^i}(u^i + v^i) + \frac{\partial}{\partial \phi^\alpha} B^{\alpha\beta}(\Omega^\beta + \omega^\beta) + \frac{\partial}{\partial v^i}(f_0^i + f_1^i) + \frac{\partial}{\partial \omega^\alpha}(J^{\alpha\beta}\Pi^\beta))(\rho W) \\ &= v^i W \frac{\partial}{\partial x^i}\rho + W\frac{\partial}{\partial \phi^\alpha}B^{\alpha\beta}\omega^\beta \rho + (u^i + v^i)\rho(\frac{v^2 - nT}{2T^2} + \frac{\omega F I \omega - nT}{2T^2})\frac{\partial T}{\partial x^i}W + (f_0^i + f_1^i)\rho(-\frac{v^i}{T})W \\ &\quad + J^{\alpha\beta}\Pi^\beta \rho(-\frac{(FI)^{\alpha\sigma}\Omega^\sigma}{T})W + \rho W \frac{\partial}{\partial \omega^\alpha}J^{\alpha\beta}\Pi^\beta\,, \end{aligned} \tag{47}$$

which can be conveniently solved in view of the relation

$$\begin{aligned} & M^\dagger(v^i W) = -\gamma(v^i W)\,,\ M^\dagger(\omega^\sigma W) = -d^\sigma(\omega^\sigma W) \\ & M^\dagger(v^i v^j - T\delta^{ij})W = -2\gamma(v^i v^j - T\delta^{ij})W\,,\ M^\dagger(\omega^\alpha\omega^\beta - TH^{\alpha\beta})W = -(d^\alpha + d^\beta)(\omega^\alpha\omega^\beta - TH^{\alpha\beta})W \\ & M^\dagger(v^i v^j v^k - T(\delta^{ij}v^k + \delta^{ik}v^j + \delta^{jk}v^i))W = -3\gamma(v^i v^j v^k - T(\delta^{ij}v^k + \delta^{ik}v^j + \delta^{jk}v^i))W \\ & M^\dagger(\omega^\alpha\omega^\beta\omega^\sigma - T(H^{\alpha\beta}\omega^\sigma + H^{\alpha\sigma}\omega^\beta + H^{\beta\sigma}\omega^\alpha))W = -(d^\alpha + d^\beta + d^\sigma)(\omega^\alpha\omega^\beta\omega^\sigma - T(H^{\alpha\beta}\omega^\sigma + H^{\alpha\sigma}\omega^\beta + H^{\beta\sigma}\omega^\alpha))W \\ & M^\dagger(v^i v^j v^k v^l - T(\delta^{ij}(v^k v^l - T\delta^{kl}) + \delta^{ik}(v^j v^l - T\delta^{jl}) + \delta^{il}(v^k v^j - T\delta^{kj}) + \delta^{kj}(v^i v^l - T\delta^{il}) \\ & \quad + \delta^{lj}(v^k v^i - T\delta^{ki}) + \delta^{lk}(v^i v^j - T\delta^{ij})) - T^2(\delta^{ij}\delta^{kl} + \delta^{ik}\delta^{jl} + \delta^{il}\delta^{kj}))W \\ & \quad = -4\gamma(v^i v^j v^k v^l - T(\delta^{ij}(v^k v^l - T\delta^{kl}) + \delta^{ik}(v^j v^l - T\delta^{jl}) + \delta^{il}(v^k v^j - T\delta^{kj}) + \delta^{kj}(v^i v^l - T\delta^{il}) \\ & \quad + \delta^{lj}(v^k v^i - T\delta^{ki}) + \delta^{lk}(v^i v^j - T\delta^{ij})) - T^2(\delta^{ij}\delta^{kl} + \delta^{ik}\delta^{jl} + \delta^{il}\delta^{kj}))W \\ & M^\dagger(\omega^\alpha\omega^\beta\omega^\sigma\omega^\rho - T(H^{\alpha\beta}\omega^\sigma\omega^\rho + H^{\alpha\sigma}\omega^\beta\omega^\rho + H^{\beta\sigma}\omega^\alpha\omega^\rho + H^{\alpha\rho}\omega^\sigma\omega^\beta \\ & \quad + H^{\beta\rho}\omega^\alpha\omega^\sigma + H^{\rho\sigma}\omega^\alpha\omega^\beta) + T^2(H^{\alpha\beta}H^{\sigma\rho} + H^{\alpha\sigma}H^{\beta\rho} + H^{\alpha\rho}H^{\beta\sigma}))W \\ & \quad = -(d^\alpha + d^\beta + d^\sigma + d^\rho)(\omega^\alpha\omega^\beta\omega^\sigma\omega^\rho - T(H^{\alpha\beta}\omega^\sigma\omega^\rho + H^{\alpha\sigma}\omega^\beta\omega^\rho + H^{\beta\sigma}\omega^\alpha\omega^\rho + H^{\alpha\rho}\omega^\sigma\omega^\beta \\ & \quad + H^{\beta\rho}\omega^\alpha\omega^\sigma + H^{\rho\sigma}\omega^\alpha\omega^\beta) + T^2(H^{\alpha\beta}H^{\sigma\rho} + H^{\alpha\sigma}H^{\beta\rho} + H^{\alpha\rho}H^{\beta\sigma}))W\,, \end{aligned} \tag{48}$$

where the dummy summation rule does not apply to d^α. From Eq. (48), it is easy to restore Eq. (12,13) in the main text with un-scaled variables. The right hand side of Eq. (47) may be broken into two parts

$$\begin{aligned} I_v &= W v^i \frac{\partial}{\partial x^i}\rho + v^i W \frac{\rho}{T}\frac{\partial T}{\partial x^i} - \frac{\rho}{T}v^i W f_0^i + u^i \rho W \frac{\partial T}{\partial x^i}(\frac{v^2 - nT}{2T^2} + \frac{\omega F I \omega - nT}{2T^2}) \\ &\quad + \frac{\rho W}{T}(v^i v^j - T\delta^{ij})\partial_j u^i + v^i \rho W \frac{\partial T}{\partial x^i}(\frac{v^2 - (n+2)T}{2T^2} + \frac{\omega F I \omega - nT}{2T^2})\,, \end{aligned} \tag{49}$$

and

$$I_\phi = W\omega^\beta \frac{\partial}{\partial \phi^\alpha}(B^{\alpha\beta}\rho) - \frac{\rho W}{T}\omega^\alpha F^{\alpha\beta}\Pi^\beta + \rho W J^{\alpha\beta}\frac{\partial}{\partial \omega^\alpha}\Pi^\beta\,. \tag{50}$$

Utilizing Eq. (48), we may invert the above two parts and obtain

$$\begin{aligned} I_v^s &= -\frac{v^i W}{\gamma T}(\frac{\partial(\rho T)}{\partial x^i} - \rho f_0^i) - \frac{u^i \rho W}{2\gamma}\frac{\partial T}{\partial x^i}\frac{v^2 - 2nT}{2T^2} - \frac{u^i \rho W}{2T^2}\frac{\partial T}{\partial x^i}\frac{(FI)^{\alpha\beta}}{d^\alpha + d^\beta}(\omega^\alpha\omega^\beta - TH^{\alpha\beta}) \\ &\quad - \frac{\rho W}{2\gamma T}(v^i v^j - T\delta^{ij})\partial_j u^i - \frac{v^i W \rho}{3\gamma}\frac{\partial T}{\partial x^i}\frac{v^2 - (n+2)T}{2T^2} - \frac{v^i \rho W}{2T^2}\frac{\partial T}{\partial x^i}\frac{(FI)^{\alpha\beta}}{d^\alpha + d^\beta + \gamma}(\omega^\alpha\omega^\beta - TH^{\alpha\beta})\,, \end{aligned} \tag{51}$$

and

$$I^s_\phi = -\frac{W\omega^\beta}{d^\beta}\partial_\alpha(B^{\alpha\beta}\rho) + \frac{\rho W}{Td^\alpha}\omega^\alpha F^{\alpha\beta}M^\beta - \frac{\rho W}{Td^\alpha}\breve{N}\varepsilon^{\alpha\beta\sigma}\omega^\alpha\Omega^\beta I^{\sigma\rho}\Omega^\rho - \frac{\rho W\omega^\alpha}{Td^\alpha}(FI)^{\alpha\beta}(\partial_t\Omega^\beta + u^i\partial_i\Omega^\beta + B^{\sigma\rho}\Omega^\rho\partial_\sigma\Omega^\beta)$$
$$- \frac{\rho W}{T(d^\alpha + d^\beta)}\breve{N}\varepsilon^{\alpha\sigma\rho}\Omega^\sigma I^{\rho\beta}(\omega^\alpha\omega^\beta - TH^{\alpha\beta}) - \frac{\rho W\omega^\alpha}{T(d^\alpha+\gamma)}(FI)^{\alpha\beta}v^i(\partial_i\Omega^\beta)$$
$$- \frac{\rho W}{T(d^\alpha + d^\beta)}(FI)^{\alpha\sigma}(\partial_\rho\Omega^\sigma)B^{\rho\beta}(\omega^\alpha\omega^\beta - TH^{\alpha\beta})\,, \tag{52}$$

where $\breve{N} = \varepsilon^{\alpha\beta\sigma}\bar{N}^{\alpha 1}\bar{N}^{\beta 2}\bar{N}^{\sigma 3} = \mathrm{Det}(\bar{N}) = 1/\mathrm{Det}(N)$. Eq. (51) and Eq. (52) give the solution of Eq. (47)

$$p^{(1)} = rW + I^s_v + I^s_\phi\,, \tag{53}$$

where the first term $r = r(x\,,\phi\,,t\,,\tau)$ is the solution for the homogeneous equation.

C. The configuration relaxation scale (ϵ)

The slow time scale is of the order ϵ, which is the configuration relaxation scale. After relaxation over the fast time scales, we have

$$M^\dagger p^{(2)} = -L^\dagger p^{(1)} + \frac{\partial}{\partial\tau}p^{(0)}\,. \tag{54}$$

By integration, the solvability condition gives

$$\begin{aligned}&\frac{\partial\rho}{\partial\tau} + \frac{\partial r}{\partial t} + \frac{\partial}{\partial x^i}(u^i r) + \frac{\partial}{\partial\phi^\alpha}(B^{\alpha\beta}\Omega^\beta r) - \frac{1}{\gamma}\frac{\partial}{\partial x^i}(\frac{\partial(\rho T)}{\partial x^i} - \rho f^i_0)\\ &- \frac{T}{d^\sigma}\frac{\partial}{\partial\phi^\alpha}B^{\alpha\beta}H^{\beta\sigma}\frac{\partial}{\partial\phi^\rho}B^{\rho\sigma}\rho + \frac{\partial}{\partial\phi^\alpha}[B^{\alpha\beta}\frac{J^{\beta\sigma}}{d^\sigma}(M^\sigma + \Pi^\sigma_0)\rho] = 0\,.\end{aligned} \tag{55}$$

The function $r(x,\phi,t,\tau)$ represents solution to the homogeneous Eq. (47), which in principle can be incorporated into function $\rho(x,\phi,t,\tau)$ and so we may set it to be zero from now on. The combination of Eq. (55) and Eq. (46) gives the total time evolution of particle density ρ. Based on this consideration, after setting the bookkeeping constant $\epsilon = 1$, we have

$$\begin{aligned}&\frac{\partial\rho}{\partial t} + \frac{\partial}{\partial x^i}(u^i\rho + \frac{f^i_0}{\gamma}\rho - \frac{\rho}{\gamma}\frac{\partial T}{\partial x^i}) + \frac{\partial}{\partial\phi^\alpha}(B^{\alpha\beta}\Omega^\beta\rho + B^{\alpha\beta}J^{\beta\sigma}(\frac{\rho}{d^\beta})(M^\sigma + \Pi^\sigma_0) - \frac{\rho T}{d^\sigma}B^{\alpha\beta}H^{\beta\sigma}\frac{\partial}{\partial\phi^\rho}B^{\rho\sigma})\\ &= \frac{\partial}{\partial x^i}\frac{T}{\gamma}\frac{\partial\rho}{\partial x^i} + \frac{T}{d^\sigma}\frac{\partial}{\partial\phi^\alpha}B^{\alpha\beta}H^{\beta\sigma}B^{\rho\sigma}\frac{\partial}{\partial\phi^\rho}\rho\,,\end{aligned} \tag{56}$$

which corresponds to the over-damped Langevin equation in the Stratonović sense

$$dX^i = (u^i + \frac{f^i_0}{\gamma} - \frac{1}{2\gamma}\frac{\partial T}{\partial x^i})dt + \sqrt{\frac{2T}{\gamma}}\circ dW^i_t \tag{57}$$

$$d\phi_\alpha = B^{\alpha\beta}(\Omega^\beta + \frac{J^{\beta\sigma}}{d^\beta}(M^\sigma + \Pi^\sigma_0) - \frac{T}{2d^\sigma}H^{\beta\sigma}\frac{\partial}{\partial\phi^\rho}B^{\rho\sigma}) + \sqrt{2T}K^{\alpha\beta}\circ d\tilde{W}^\beta_t\,, \tag{58}$$

where $K^{\alpha\beta} = B^{\alpha\sigma}C^{\sigma\beta}$, $(C^2)^{\alpha\beta} = H^{\alpha\beta}/d^\beta$. Eq. (57) corresponds to Eq. (8) and Eq. (58) to Eq. (14) in the main text which does not have the fluid rotation term. Nevertheless, the local charts of orientations are used in the main text which removes one term in Eq. (58). In this sense, the local chart representation is simple. However, it is valid only locally. The Euler angle description Eq. (58) is valid globally.

D. The entropy production in the over-damped case

It is convenient to directly evoke Eq. (27) to compute entropy production in the environment associated with Eq. (56). To utilize the equation, from Eq. (56), with the usual time-reversal operation we identify

$$\begin{aligned}
&\hat{V}^i_+ = \frac{1}{\gamma}(f^i_0 - \frac{\partial T}{\partial x^i})\,,\ V^i_- = u^i \\
&\hat{\omega}^\alpha_+ = \frac{1}{d^\beta}B^{\alpha\beta}J^{\beta\sigma}(M^\sigma + \Pi^\sigma_0) - \frac{T}{d^\sigma}B^{\alpha\beta}H^{\beta\sigma}(\frac{\partial}{\partial\phi^\rho}B^{\rho\sigma})\,,\ \omega^\alpha_- = B^{\alpha\beta}\Omega^\beta\,.
\end{aligned}$$

So, in the over-damped limit, the entropy production is

$$\begin{aligned}
\hat{S}^{\text{(over)}}_{\text{env}} = &\int \frac{1}{T}(f^i_0 - \frac{\partial T}{\partial x^i})(\circ dx^i - u^i dt) + \int \frac{1}{T}[\frac{1}{d^\mu}B^{\alpha\mu}J^{\mu\sigma}(M^\sigma + \Pi^\sigma_0) - \frac{T}{d^\sigma}B^{\alpha\mu}H^{\mu\sigma}(\frac{\partial B^{\rho\sigma}}{\partial\phi^\rho})] \\
&C^{\alpha\beta}(\circ d\phi^\beta - B^{\beta\sigma}\Omega^\sigma dt) - \int \partial_\alpha(B^{\alpha\beta}\Omega^\beta)dt\,,
\end{aligned} \tag{59}$$

where $C^{-1}_{\alpha\beta} = B^{\alpha\sigma}\frac{H^{\sigma\rho}}{d^\rho}B^{\beta\rho}$. Further calculation converts Eq. (59) to

$$\hat{S}^{\text{(over)}}_{\text{env}} = \int \frac{dt}{T}(f^i_0 - \frac{\partial T}{\partial x^i})v^i + (M^\alpha + \Pi^\alpha_0)F^{\alpha\beta}\omega^\beta + \int dt\frac{\partial B^{\alpha\beta}}{\partial\phi^\alpha}\Omega^\beta - \frac{\partial}{\partial\phi^\alpha}(B^{\alpha\beta}\Omega^\beta) + \int \frac{d\theta}{\tan\theta}\,. \tag{60}$$

The last term is a geometric factor accounting for the metric change due to the nonlinear coordinate system that we use. The second and third last terms seem to describe entropy production of the fluid itself due to its rotation, which vanish for the irrotational flow.

Compared with Eq. (31), the kinetic energy terms are missing in Eq. (60), which is reasonable in the large friction limit since the inertia terms are essentially thrown out of the dynamics in this limit. Below, we will compute the ensemble averages of various terms of Eq. (31) to show their relevance.

IV. THE AVERAGE ENTROPY PRODUCTION BASED ON THE FEYNMANN-KAC FORMULA

To compute averages along an orbit, Feyman-Kac formula is used to derive an equation for the generating function. In the limit of small inertia, this equation can be solved with the multiple scale analysis to give the solvability condition from which the time change rate of the averages could be derived. We start from Eq. (31), which defines the entropy produced in the environment and can be rewritten in the following form

$$\begin{aligned}
\hat{S}_{\text{env}} = &-\ln W_{t'} + \ln W_t + \int_{t'}^{t} \frac{dt}{T}(f^i_0 - \frac{\partial T}{\partial x^i})v^i + (M^\alpha + \Pi^\alpha_0)F^{\alpha\beta}\omega^\beta + (T\delta^{ij} - v^i v^j)\partial_i u^j \\
&+\frac{(nT - v^i v^i) + (nT - \omega^\alpha (FI)^{\alpha\beta}\omega^\beta)}{2T}(\frac{\partial T}{\partial t} + u^i\frac{\partial T}{\partial x^i}) + \check{N}\varepsilon^{\alpha\beta\sigma}\Omega^\beta I^{\sigma\rho}(TH^{\alpha\rho} - \omega^\alpha\omega^\rho) \\
&+(FI)^{\alpha\beta}(\partial_\sigma\Omega^\beta)B^{\sigma\rho}(TH^{\alpha\rho} - \omega^\alpha\omega^\rho) - (FI)^{\alpha\beta}\omega^\alpha(\partial_i\Omega^\beta)v^i + \frac{(n+2)T - v^2}{2T}v^i(\partial_i T) + \frac{nT - \omega FI\omega}{2T}v^i(\partial_i T)\,,
\end{aligned} \tag{61}$$

where

$$\ln W = -\frac{v^i v^i}{2T} - \frac{\omega^\alpha (FI)^{\alpha\beta}\omega^\beta}{2T} - n\ln 2\pi T + \frac{1}{2}\ln \text{Det}(FI)\,. \tag{62}$$

Eq. (62) itself is related to the entropy of the Brownian particle defined in Eq. (34). Eq. (61) leads to Eq. (9) and (15) in the main text. The stochastic integral in Eq. (61) may be split into three parts

$$S_{\text{env}} = -\ln W_{t'} + \ln W_t + S_{\text{reg}} + S_{\text{quad}} + S_{\text{cube}}\,, \tag{63}$$

where $S_{\rm reg}$ is the regular part of the entropy production which already appears in the large dissipation limit Eq. (59); $S_{\rm quad}$ represents the part induced by the time change of the temperature and the fluid flow. $S_{\rm cube}$ originates from the spatial variation of the temperature and the kinetic energy of the particle. More explicitly, their definitions are listed below

$$S_{\rm reg} = \int_{t'}^{t} \frac{dt}{T}(f_0^i - \frac{\partial T}{\partial x^i})v^i + (M^\alpha + \Pi_0^\alpha)F^{\alpha\beta}\omega^\beta \tag{64}$$

$$S_{\rm quad} = \int_{t'}^{t} \frac{dt}{T}(T\delta^{ij} - v^i v^j)\partial_i u^j + \frac{(nT - v^i v^i) + (nT - \omega^\alpha (FI)^{\alpha\beta}\omega^\beta)}{2T}(\frac{\partial T}{\partial t} + u^i \frac{\partial T}{\partial x^i})$$
$$+\check{N}\varepsilon^{\alpha\beta\sigma}\Omega^\beta I^{\sigma\rho}(TH^{\alpha\rho} - \omega^\alpha\omega^\rho) + (FI)^{\alpha\beta}(\partial_\sigma \Omega^\beta)B^{\sigma\rho}(TH^{\alpha\rho} - \omega^\alpha\omega^\rho) - (FI)^{\alpha\beta}\omega^\alpha(\partial_i\Omega^\beta)v^i \tag{65}$$

$$S_{\rm cube} = \int_{t'}^{t} \frac{dt}{T}\frac{(n+2)T - v^2}{2T}v^i(\partial_i T) + \frac{nT - \omega F I \omega}{2T}v^i(\partial_i T)\,, \tag{66}$$

where the integrands in the above three integrals are proportional to the first, second and third-order Hermite polynomials. The joint generating function of the three contribution is

$$G_s(x\,,\phi\,,v\,,\omega\,,t|x'\,,\phi'\,,v'\,,\omega'\,,t') = \langle \exp(-s_1 S_{\rm reg} - s_2 S_{\rm quad} - s_3 S_{\rm cube})\delta(x_t - x)\delta(\phi_t - \phi)\delta(v_t - v)\delta(\omega_t - \omega)\rangle\,, \tag{67}$$

where the average is taken over the paths with fixed initial conditions $(x'\,,\phi'\,,v'\,,\omega')$ at time t' and the δ-function selects the final conditions to be $(x\,,\phi\,,v\,,\omega)$ at time t. Eq. (67) obeys the forward Feynman-Kac formula [1]

$$\begin{aligned}\partial_t G_s - L_0^\dagger G_s = -\Bigg[& s_1((f_0^i - \frac{\partial T}{\partial x^i})\frac{v^i}{T} + (M^\alpha + \Pi_0^\alpha)F^{\alpha\beta}\frac{\omega^\beta}{T}) + \frac{s_2}{T}((T\delta^{ij} - v^i v^j)\partial_i u^j \\ &+ \frac{(nT - v^i v^i) + (nT - \omega^\alpha (FI)^{\alpha\beta}\omega^\beta)}{2T}(\frac{\partial T}{\partial t} + u^i \frac{\partial T}{\partial x^i}) + \check{N}\varepsilon^{\alpha\beta\sigma}\Omega^\beta I^{\sigma\rho}(TH^{\alpha\rho} - \omega^\alpha\omega^\rho) \\ &+ (FI)^{\alpha\beta}(\partial_\sigma\Omega^\beta)B^{\sigma\rho}(TH^{\alpha\rho} - \omega^\alpha\omega^\rho) - (FI)^{\alpha\beta}\omega^\alpha(\partial_i\Omega^\beta)v^i) \\ &+ \frac{s_3}{T}(\frac{(n+2)T - v^2}{2T}v^i(\partial_i T) + \frac{nT - \omega F I \omega}{2T}v^i(\partial_i T))\Bigg] G_s\end{aligned}\,, \tag{68}$$

where

$$L_0 = (u^i + v^i)\frac{\partial}{\partial x^i} + B^{\alpha\beta}(\omega^\beta + \Omega^\beta)\frac{\partial}{\partial \phi^\alpha} + (f_0^i + f_1^i)\frac{\partial}{\partial v^i} - \gamma v^i \frac{\partial}{\partial v^i} + J^{\alpha\beta}\Pi^\beta \frac{\partial}{\partial \phi^\alpha} - D^{\alpha\beta}\omega^\beta\frac{\partial}{\partial \omega^\alpha} + T\gamma\frac{\partial^2}{\partial v^i \partial v^i} + TS^{\alpha\beta}\frac{\partial^2}{\partial \omega^\alpha \partial\omega^\beta}\,. \tag{69}$$

The multi-scale analysis similar to what we did in previous sections is used to analyze Eq. (68). Introducing the fast time θ, the slow time τ and the scaling exactly like that in Eq. (36), we have

$$(\epsilon^{-1}\frac{\partial}{\partial\theta} + \frac{\partial}{\partial t} + \epsilon\frac{\partial}{\partial\tau})G_s - (L^\dagger + \epsilon^{-1}M^\dagger - \epsilon s_2 \frac{(nT - v^i v^i) + (nT - \omega^\alpha (FI)^{\alpha\beta}\omega^\beta)}{2T^2}\frac{\partial T}{\partial \tau})G_s = 0\,, \tag{70}$$

where

$$\begin{aligned}-L^\dagger =& \frac{\partial}{\partial x^i}(u^i + v^i) + \frac{\partial}{\partial\phi^\alpha}B^{\alpha\beta}(\omega^\beta + \Omega^\beta) + \frac{\partial}{\partial v^i}(f_0^i + f_1^i) + \frac{\partial}{\partial\omega^\alpha}(J^{\alpha\beta}\Pi^\beta) + s_1((f_0^i - \frac{\partial T}{x^i})\frac{v^i}{T} + (M^\alpha + \Pi_0^\alpha)F^{\alpha\beta}\frac{\omega^\beta}{T}) \\ &+ \frac{s_2}{T}((T\delta^{ij} - v^i v^j)\partial_i u^j + \frac{(nT - v^i v^i) + (nT - \omega^\alpha (FI)^{\alpha\beta}\omega^\beta)}{2T}(u^i\frac{\partial T}{\partial x^i}) + \check{N}\varepsilon^{\alpha\beta\sigma}\Omega^\beta I^{\sigma\rho}(TH^{\alpha\rho} - \omega^\alpha\omega^\rho) \\ &+ (FI)^{\alpha\beta}(\partial_\sigma\Omega^\beta)B^{\sigma\rho}(TH^{\alpha\rho} - \omega^\alpha\omega^\rho) - (FI)^{\alpha\beta}\omega^\alpha(\partial_i\Omega^\beta)v^i) + \frac{s_3}{T}(\frac{(n+2)T - v^2}{2T}v^i(\partial_i T) + \frac{nT - \omega F I\omega}{2T}v^i(\partial_i T)) \\ M^\dagger =& \gamma\left[\frac{\partial}{\partial v^i}v^i + T\frac{\partial^2}{\partial v^i\partial v^i}\right] + \left[\frac{\partial}{\partial\omega^\alpha}D^{\alpha\beta}\omega^\beta + TS^{\alpha\beta}\frac{\partial^2}{\partial\omega^\alpha\partial\omega^\beta}\right]\,.\end{aligned}$$

Terms involving $\partial T/\partial\tau$ appear in Eq. (70) since the temperature is assumed to be a slow variable of time. Note that here the definition of $L^\dagger$ is consistent with the previous one, which will bring much convenience to later perturbative solution of Eq. (70). Proceeding as before by assuming

$$G_s = G^{(0)} + \epsilon G^{(1)} + \epsilon^2 G^{(2)} + \cdots \,, \tag{71}$$

we substitute Eq. (71) into Eq. (70) and compare the coefficients of different orders of ϵ, which results in the equations satisfied by $G^{(0)}\,, G^{(1)}\,, G^{(2)}\,, \cdots$.
At the order ϵ^{-1}, we have

$$(\frac{\partial}{\partial\theta} - M^\dagger)G^{(0)} = 0\,, \tag{72}$$

which, after relaxation over the fast time scale, results in

$$G^{(0)} = q(x\,,\phi\,,t\,,\tau)W(v\,,\omega|x\,,\phi\,,\tau)\,. \tag{73}$$

At the order ϵ^0, we have

$$(\frac{\partial}{\partial\theta} - M^\dagger)G^{(1)} = -(\frac{\partial}{\partial t} - L^\dagger)G^{(0)}\,. \tag{74}$$

After the initial fast transient relaxation dynamics, based on Eq. (74), the solvability condition gives

$$\frac{\partial q}{\partial t} + \frac{\partial}{\partial x^i}(u^i q) + \frac{\partial}{\partial\phi^\alpha}(B^{\alpha\beta}\Omega^\beta q) = 0\,, \tag{75}$$

which is the continuity equation for q at the lowest order. Eq. (74) then becomes

$$\begin{aligned} M^\dagger G^{(1)} = \Bigg[& v^i\frac{\partial q}{\partial x^i} + \omega^\beta\frac{\partial}{\partial\phi^\alpha}(B^{\alpha\beta}q) + (s_1-1)q((f_0^i - \frac{\partial T}{x^i})\frac{v^i}{T} + (M^\alpha + \Pi_0^\alpha)F^{\alpha\beta}\frac{\omega^\beta}{T}) \\ & + \frac{(s_2-1)q}{T}((T\delta^{ij} - v^iv^j)\partial_i u^j + \frac{(nT - v^iv^i) + (nT - \omega^\alpha(FI)^{\alpha\beta}\omega^\beta)}{2T}(u^i\frac{\partial T}{\partial x^i}) \\ & + \check{N}\varepsilon^{\alpha\beta\sigma}\Omega^\beta I^{\sigma\rho}(TH^{\alpha\rho} - \omega^\alpha\omega^\rho) + (FI)^{\alpha\beta}(\partial_\sigma\Omega^\beta)B^{\sigma\rho}(TH^{\alpha\rho} - \omega^\alpha\omega^\rho) - (FI)^{\alpha\beta}\omega^\alpha(\partial_i\Omega^\beta)v^i) \\ & + \frac{(s_3-1)q}{T}(\frac{(n+2)T - v^2}{2T}v^i(\partial_i T) + \frac{nT - \omega FI\omega}{2T}v^i(\partial_i T))\Bigg] W \end{aligned}\,, \tag{76}$$

which has a solution

$$\begin{aligned} G^{(1)} = \Bigg[& -\frac{v^i}{\gamma}\frac{\partial q}{\partial x^i} - \frac{\omega^\beta}{d^\beta}\frac{\partial}{\partial\phi^\alpha}(B^{\alpha\beta}q) + (s_1-1)q(-\frac{1}{\gamma}(f_0^i - \frac{\partial T}{x^i})\frac{v^i}{T} - (M^\alpha + \Pi_0^\alpha)F^{\alpha\beta}\frac{\omega^\beta}{d^\beta T}) \\ & + \frac{(s_2-1)q}{T}(-\frac{1}{2\gamma}(T\delta^{ij} - v^iv^j)\partial_i u^j - \frac{1}{2\gamma}\frac{(nT - v^iv^i)}{2T}(u^j\frac{\partial T}{\partial x^j}) - \frac{(FI)^{\alpha\beta}}{2T(d^\alpha + d^\beta)}(TH^{\alpha\beta} - \omega^\alpha\omega^\beta)(u^i\frac{\partial T}{\partial x^i}) \\ & - (\check{N}\varepsilon^{\alpha\beta\sigma}\Omega^\beta I^{\sigma\rho} + (FI)^{\alpha\beta}(\partial_\sigma\Omega^\beta)B^{\sigma\rho})\frac{TH^{\alpha\rho} - \omega^\alpha\omega^\rho}{d^\alpha + d^\rho} + \frac{(FI)^{\alpha\beta}\omega^\alpha(\partial_i\Omega^\beta)v^i}{\gamma + d^\alpha}) \\ & + \frac{(s_3-1)q}{2T^2}(-\frac{1}{3\gamma}((n+2)T - v^2)v^i(\partial_i T) - v^i(\partial_i T)\frac{(FI)^{\beta\sigma}}{d^\beta + d^\sigma + \gamma}(\omega^\beta\omega^\sigma - TH^{\beta\sigma})\Bigg] W\,. \end{aligned} \tag{77}$$

At the order ϵ^1, after relaxation, we have

$$M^\dagger G^{(2)} = \frac{\partial G^{(0)}}{\partial\tau} + s_2\frac{(nT - v^iv^i) + (nT - \omega^\alpha(FI)^{\alpha\beta}\omega^\beta)}{2T^2}\frac{\partial T}{\partial\tau}G^{(0)} - L^\dagger G^{(1)} + \frac{\partial}{\partial t}G^{(1)}\,. \tag{78}$$

The solvability condition implies

$$\begin{aligned}
&\frac{\partial q}{\partial \tau}-\frac{\partial}{\partial x^i}\frac{T}{\gamma}\frac{\partial q}{\partial x^i}-(s_1-1)\frac{\partial}{\partial x^i}(\frac{f_0^i}{T}-\frac{1}{T}\frac{\partial T}{\partial x^i})\frac{Tq}{\gamma}-\frac{T}{d^\beta}\frac{\partial}{\partial \phi^\sigma}B^{\sigma\rho}H^{\rho\beta}\frac{\partial}{\partial \phi^\alpha}B^{\alpha\beta}q-\frac{s_1}{\gamma}(f_0^i-\frac{\partial T}{\partial x^i})\frac{\partial q}{\partial x^i}\\
&-s_1(s_1-1)\frac{qT}{\gamma}(\frac{f_0^i}{T}-\frac{1}{T}\frac{\partial T}{\partial x^i})(\frac{f_0^i}{T}-\frac{1}{T}\frac{\partial T}{\partial x^i})-\frac{s_1-1}{d^\beta}\frac{\partial}{\partial \phi^\sigma}B^{\sigma\rho}H^{\rho\beta}(M^\alpha+\Pi_0^\alpha)F^{\alpha\beta}q\\
&-\frac{s_1(s1-1)}{d^\beta T}(M^\sigma+\Pi_0^\sigma)F^{\sigma\rho}H^{\rho\beta}(M^\alpha+\Pi_0^\alpha)F^{\alpha\beta}q-\frac{s_1}{d^\beta}(M^\sigma+\Pi_0^\sigma)F^{\sigma\rho}H^{\rho\beta}\frac{\partial}{\partial\phi^\alpha}(B^{\alpha\beta}q)\\
&+\frac{s_2-s_2^2}{2\gamma}q(\partial_j u^i\partial_i u^j+\partial_j u^i\partial_j u^i)+\frac{s_2-s_2^2}{4\gamma T^2}nq(u^i\partial_i T)^2+\frac{s_2-s_2^2}{4T^2}(u^i\frac{\partial T}{\partial x^i})^2 q\bar{d}_{jj}+\frac{s_2-s_2^2}{Td^\beta}q(u^i\frac{\partial T}{\partial x^i})(\partial_\sigma\Omega^\beta)B^{\sigma\beta}\\
&+\frac{s_2-s_2^2}{Td^\alpha}\breve{N}q(u^i\frac{\partial T}{\partial x^i})\varepsilon^{\alpha\beta\sigma}\Omega^\beta\bar{F}^{\sigma\alpha}+\frac{s_2-s_2^2}{(d^\alpha+d^\rho)}q\breve{N}^2\varepsilon^{\alpha\beta\sigma}\Omega^\beta\bar{F}^{\sigma\rho}(S/D)^\alpha\Omega^\mu(\varepsilon^{\alpha\mu\nu}I^{\nu\rho}+\varepsilon^{\rho\mu\nu}I^{\nu\alpha})\\
&+\frac{2(s_2-s_2^2)q}{d^\alpha+d^\rho}\breve{N}\varepsilon^{\alpha\beta\sigma}\Omega^\beta I^{\sigma\rho}((\partial_\mu\Omega^\alpha)B^{\mu\rho}(S/D)^\rho+(\partial_\mu\Omega^\rho)B^{\mu\alpha}(S/D)^\alpha)\\
&+\frac{(s_2-s_2^2)q}{(d^\alpha+d^\rho)}(\partial_\beta\Omega^\alpha)B^{\beta\rho}((S/D)^\rho(D/S)^\alpha(\partial_\sigma\Omega^\alpha)B^{\sigma\rho}+(\partial_\sigma\Omega^\rho)B^{\sigma\alpha})+\frac{(s_2-s_2^2)q}{\gamma+d^\alpha}(FI)^{\alpha\beta}(\partial_i\Omega^\beta)(\partial_i\Omega^\alpha)\\
&+\frac{(s_3-s_3^2)q}{6\gamma T}\left(\frac{\partial T}{\partial x^i}\right)^2(n+2)+\frac{(s_3-s_3^2)q}{2T}\bar{c}_{jj}\left(\frac{\partial T}{\partial x^i}\right)^2=0
\end{aligned}, \tag{79}$$

which by setting $\epsilon=1$ and combined with Eq. (75) gives

$$\begin{aligned}
&\frac{\partial q}{\partial t}+\frac{\partial}{\partial x^i}(u^i q)+\frac{\partial}{\partial\phi^\alpha}(B^{\alpha\beta}\Omega^\beta q)-\frac{\partial}{\partial x^i}\frac{T}{\gamma}\frac{\partial q}{\partial x^i}-(s_1-1)\frac{\partial}{\partial x^i}(\frac{f_0^i}{T}-\frac{1}{T}\frac{\partial T}{\partial x^i})\frac{Tq}{\gamma}-\frac{T}{d^\beta}\frac{\partial}{\partial \phi^\sigma}B^{\sigma\rho}H^{\rho\beta}\frac{\partial}{\partial \phi^\alpha}B^{\alpha\beta}q\\
&-\frac{s_1}{\gamma}(f_0^i-\frac{\partial T}{\partial x^i})\frac{\partial q}{\partial x^i}-s_1(s_1-1)\frac{qT}{\gamma}(\frac{f_0^i}{T}-\frac{1}{T}\frac{\partial T}{\partial x^i})(\frac{f_0^i}{T}-\frac{1}{T}\frac{\partial T}{\partial x^i})-\frac{s_1-1}{d^\beta}\frac{\partial}{\partial \phi^\sigma}B^{\sigma\rho}H^{\rho\beta}(M^\alpha+\Pi_0^\alpha)F^{\alpha\beta}q\\
&-\frac{s_1(s1-1)}{d^\beta T}(M^\sigma+\Pi_0^\sigma)F^{\sigma\rho}H^{\rho\beta}(M^\alpha+\Pi_0^\alpha)F^{\alpha\beta}q-\frac{s_1}{d^\beta}(M^\sigma+\Pi_0^\sigma)F^{\sigma\rho}H^{\rho\beta}\frac{\partial}{\partial\phi^\alpha}(B^{\alpha\beta}q)\\
&+\frac{s_2-s_2^2}{2\gamma}q(\partial_j u^i\partial_i u^j+\partial_j u^i\partial_j u^i)+\frac{s_2-s_2^2}{4\gamma T^2}nq(u^i\partial_i T)^2+\frac{s_2-s_2^2}{4T^2}(u^i\frac{\partial T}{\partial x^i})^2 q\bar{d}_{jj}+\frac{s_2-s_2^2}{Td^\beta}q(u^i\frac{\partial T}{\partial x^i})(\partial_\sigma\Omega^\beta)B^{\sigma\beta}\\
&+\frac{s_2-s_2^2}{Td^\alpha}\breve{N}q(u^i\frac{\partial T}{\partial x^i})\varepsilon^{\alpha\beta\sigma}\Omega^\beta\bar{F}^{\sigma\alpha}+\frac{s_2-s_2^2}{(d^\alpha+d^\rho)}q\breve{N}^2\varepsilon^{\alpha\beta\sigma}\Omega^\beta\bar{F}^{\sigma\rho}(S/D)^\alpha\Omega^\mu(\varepsilon^{\alpha\mu\nu}I^{\nu\rho}+\varepsilon^{\rho\mu\nu}I^{\nu\alpha})\\
&+\frac{2(s_2-s_2^2)q}{d^\alpha+d^\rho}\breve{N}\varepsilon^{\alpha\beta\sigma}\Omega^\beta I^{\sigma\rho}((\partial_\mu\Omega^\alpha)B^{\mu\rho}(S/D)^\rho+(\partial_\mu\Omega^\rho)B^{\mu\alpha}(S/D)^\alpha)\\
&+\frac{(s_2-s_2^2)q}{(d^\alpha+d^\rho)}(\partial_\beta\Omega^\alpha)B^{\beta\rho}((S/D)^\rho(D/S)^\alpha(\partial_\sigma\Omega^\alpha)B^{\sigma\rho}+(\partial_\sigma\Omega^\rho)B^{\sigma\alpha})+\frac{(s_2-s_2^2)q}{\gamma+d^\alpha}(FI)^{\alpha\beta}(\partial_i\Omega^\beta)(\partial_i\Omega^\alpha)\\
&+\frac{(s_3-s_3^2)q}{6\gamma T}\left(\frac{\partial T}{\partial x^i}\right)^2(n+2)+\frac{(s_3-s_3^2)q}{2T}\bar{c}_{jj}\left(\frac{\partial T}{\partial x^i}\right)^2=0
\end{aligned}, \tag{80}$$

where $\bar{d}_{jj}=\sum_j 1/d^j\,,\bar{c}_{jj}=\sum_j 1/(2d^j+\gamma)$ and $(S/D)_\alpha$ and $(D/S)_\alpha$ are diagonal entries of the diagonal matrices $SD^{-1}\,,DS^{-1}$.

V. DISCUSSION

Concerning Eq. (80), we have the following remarks:

- In the equation, s_2 does appear and is fully associated with the fluid velocity u^i. As before, The slow time variation of the temperature does not contribute to the entropy production at this order.
- Setting $s_2 = 0\,, s_3 = 0$, the generating equation on over-damped trajectories will give

$$S_{\mathrm{reg}} = \int_{t'}^{t} \frac{dt}{T}(f_0^i - \frac{\partial T}{\partial x^i})v^i + (M^\alpha + \Pi_0^\alpha)F^{\alpha\beta}\omega^\beta \to \hat{S}_{\mathrm{env}}^{(\mathrm{over})}\,. \tag{81}$$

Therefore, the terms multiplied by s_2 and s_3 cannot be associated to any sequential over-damped functional and may be regarded as anomalous entropy production. In particular, if $f = 0\,, u = 0$, the average entropy production rate could be derived directly as

$$\begin{aligned}\frac{d}{dt}\langle S_{\mathrm{reg}}\rangle &= -\frac{d}{dt}\int d^3x d^3\phi d^3v d^3\omega \frac{\partial G_s}{\partial s_1}|_{s=0} \\ &= \int d^3x d^3\phi \frac{1}{\gamma T}\frac{\partial T}{\partial x^i}\frac{\partial Tq}{\partial x^i}\,.\end{aligned} \tag{82}$$

With the particle entropy $S_p = -\ln q$, the total entropy production in this case is

$$\frac{d}{dt}S_{\mathrm{tot}} = \int d^3x d^3\phi \frac{|\nabla(qT)|^2}{\gamma q T}\,, \tag{83}$$

which is exactly what is obtained in [1].

- If there is no average flow, *i.e.* $u^i = 0$, the average anomalous entropy production rate is given by

$$\begin{aligned}\frac{d}{dt}\langle S_{\mathrm{cube}}\rangle &= -\frac{d}{dt}\int d^3x d^3\phi d^3v d^3\omega \frac{\partial G_s}{\partial s_3}|_{s=0} \\ &= \int d^3x d^3\phi \frac{q}{6\gamma T}\left(\frac{\partial T}{\partial x^i}\right)^2 (n+2) + \frac{q}{2T}\bar{c}_{jj}\left(\frac{\partial T}{\partial x^i}\right)^2\,,\end{aligned} \tag{84}$$

which in form is surprisingly close to what we obtained before [1]. The first term is associated with translation while the second one is related to rotation. In particular, when the Brownian particle is spherical, we recovered the previous result since $\bar{c}_{jj} = \sum_j 1/(2d^j + \gamma) \to n/(2\gamma_2 + \gamma)$ as all $d^j \to \gamma_2$. Here, in general, the d^j's could be different, which brings the expected complication to the case with non-spherical Brownian particle though the resulting expression is unexpectedly simple. So far, all the variables in the integrands are in the scaled form. If we re-scale them back to the original variables, Eq. (84) keeps exactly the same form when the time derivative is also re-scaled back except that now we write $\bar{c}_{jj} = \sum_j 1/(2md^j + \gamma)$ where m is the particle mass, in accordance with the entropy production term below Eq. (16) in the main text.

- The average flow seems to bring quite many terms into the entropy production computation. For an irrotational flow, $\Omega^j = 0$, the entropy produced due to the flow could be written as

$$\begin{aligned}\frac{d}{dt}\langle S_{\mathrm{quad}}\rangle &= -\frac{d}{dt}\int d^3x d^3\phi d^3v d^3\omega \frac{\partial G_s}{\partial s_2}|_{s=0} \\ &= \int d^3x d^3\phi \frac{1}{2\gamma}q(\partial_j u^i \partial_i u^j + \partial_j u^i \partial_j u^i) + \frac{1}{4\gamma T^2}nq(\partial_t T + u^i\partial_i T)^2 \\ &\quad + \frac{1}{4T^2}(\partial_t T + u^i \partial_i T)^2 q\bar{d}_{jj}\,,\end{aligned} \tag{85}$$

which is easily seen to be positive if $\bar{d}_{jj} = \mathrm{Tr}(J\Gamma) > 0$. To keep the Galilean invariance, we add the higher-order term $\partial_t T$ in Eq. (85). As we assumed the slow time variation of the temperature $T = T(x, \tau)$, the extra terms disappear in the averaging process and will only appear in the later higher-order terms. If we re-scale all the variables back to the original one, the mass m should be multiplied with the integrand, which leads to Eq. (3) and the mixed term $\int \frac{1}{4T^2}|\partial_t T + u\cdot\partial_x T|^2 \frac{mn}{\gamma}$ in the advection-diffusion limit in the main text.

In the overdamped limit, Langevin equation is considered to be a very good description of Brownian motion. However, a previous paper [1] shows the existence of anomalous entropy production which cannot be accounted for by the Langevin equation. Here, we studied the stochastic thermodynamics of a rotating Brownian particle in a gradient fluid flow and found many extra terms which contribute to the anomalous entropy production. Also, in the current or previous treatment, we neglect the back reaction of the induced hydrodynamic flow on the particle itself. When the fluid is not extremely viscous, the fluid flow induced by the particle motion has a non-negligible effect on particles within a certain range, this coupling certainly will change the particle behavior and hence the energy flow and entropy production.

[1] Celani, A., Bo, S., Eichhorn, R. & Aurell, E. Anomalous thermodynamics at the micro-scale. *Phys. Rev. Lett.* **109** (2012).

[2] Bustamante, C., Liphardt, J. & Ritort, F. The nonequilibrium thermodynamics of small systems. *Physics Today* **58**, 43 (2005).

[3] Chakrabarty, A. *et al.* Brownian motion of boomerang colloidal particles. *Phys. Rev. Lett.* **111** (2013).

[4] Han, Y. *et al.* Brownian motion of an ellipsoid. *Science* **314** (2006).

[5] Cichocki, B., E-Jeewska, M. L. & Wajnryb, E. Translational brownian motion for particles of arbitrary shape. *J. Chem. Phys.* **136** (2012).

[6] Chetrite, R. & Gawędzki, K. Fluctuation relations for diffusion processes. *Commun. Math. Phys.* **282**, 469–518 (2007). 0707.2725.

[7] Seifert, U. Entropy production along a stochastic trajectory and an integral fluctuation theorem. *Phys. Rev. Lett.* **95**, 040602 (2005).
